# Changes in the Fecal Metabolome Are Associated with Feeding Fiber Not Health Status in Cats with Chronic Kidney Disease

**DOI:** 10.3390/metabo10070281

**Published:** 2020-07-09

**Authors:** Jean A. Hall, Dennis E. Jewell, Eden Ephraim

**Affiliations:** 1 Department of Biomedical Sciences, College of Veterinary Medicine, Oregon State University, Corvallis, OR 97333-4802, USA; 2 Department of Grain Science and Industry, Kansas State University, Manhattan, KS 66506, USA; djewell@ksu.edu; 3 Pet Nutrition Center, Hill’s Pet Nutrition, Topeka, KS 66617-1587, USA; eden_ephraim_gebreselassie@hillspet.com

**Keywords:** apple pomace, betaine, cats, chronic kidney disease, dietary fiber, fecal metabolites, metabolomics, oat beta glucan, short chain fructooligosaccharides

## Abstract

The objective was to determine the effects of feeding different fiber sources to cats with chronic kidney disease (CKD) compared with healthy cats (both n = 10) on fecal metabolites. A cross-over within split-plot study design was performed using healthy and CKD cats (IRIS stage 1, 2, and 3). After cats were fed a complete and balanced dry food designed to aid in the management of renal disease for 14 days during a pre-trial period, they were randomly assigned to two fiber treatments for 4 weeks each. The treatment foods were formulated similar to pre-trial food and contained 0.500% betaine, 0.586% oat beta glucan, and either 0.407% short chain fructooligosaccharides (scFOS) fiber or 3.44% apple pomace. Both treatment foods had similar crude fiber (2.0 and 2.1% for scFOS and apple pomace, respectively) whereas soluble fiber was 0.8 and 1.6%, respectively. At baseline, CKD had very little impact on the fecal metabolome. After feeding both fiber sources, some fecal metabolite concentrations were significantly different compared with baseline. Many fecal uremic toxins decreased, although in healthy cats some increased; and some more so when feeding apple pomace compared with scFOS, e.g., hippurate, 4-hydroxyhippurate, and 4-methylcatechol sulfate; the latter was also increased in CKD cats. Changes in secondary bile acid concentrations were more numerous in healthy compared with CKD cats, and cats in both groups had greater increases in some secondary bile acids after consuming apple pomace compared with scFOS, e.g., tauroursodeoxycholate and hyocholate. Although changes associated with feeding fiber were more significant than changes associated with disease status, differential modulation of the gut-kidney axis using dietary fiber may benefit cats.

## 1. Introduction

Chronic kidney disease (CKD) is a major cause of morbidity in cats [1]. The goal of our studies is to modify currently available maintenance foods with alternative ingredients to delay the decline in kidney function and loss of lean body mass in cats with CKD [2]. We have previously shown that feeding foods containing betaine and prebiotics significantly increases total body mass in cats with CKD [3]. Betaine, named after its discovery in sugar beets, is a small N-trimethylated amino acid that serves as an organic osmolyte and methyl donor. It is taken up by cells for protection against osmotic stress, and involved in methylation reactions and the detoxification of homocysteine. In humans and mice, feeding betaine has been shown to alleviate osmotic stress and the accumulation of homocysteine [4,5]. Prebiotics are indigestible ingredients that favor the growth of certain bacteria. With kidney disease, impaired protein assimilation in the proximal intestine results in excess protein load delivered to the colon, and leads to the proliferation of proteolytic bacteria at the expense of saccharolytic bacteria [6]. Excessive uremic toxins are produced as a result of the altered gut microbial composition (the gut-kidney axis) [7]. End-stage kidney disease is characterized by the retention of solutes referred to as uremic toxins. The origin of these solutes could be either from endogenous host metabolism or the gut microbiome [8]. Targeted interventions may be beneficial for re-establishing symbiosis, e.g., prebiotics favor the growth of saccharolytic bacteria in the gut over putrefactive bacteria [7].

Microbial fermentation of dietary fiber in the hindgut of cats is considered important for health even though cats are obligate carnivores [9]. The effects of feeding fiber to cats with CKD, as well as the best type of fiber to feed, have not been well studied. Because the type of fermentable fiber fed influences fecal microbial composition [10], we hypothesized that concentrations of microbiota-generated toxins and their precursor metabolites may be altered in the feces.

The objective of this study was to evaluate the effects of feeding betaine, oat beta glucan, and either short chain fructooligosaccharides (scFOS) or apple pomace as additional fiber sources to healthy cats and cats with CKD, International Renal Interest Society (IRIS) stages 1, 2, and 3 [11], on the fecal metabolome. Fecal metabolomics provides direct insight into the gut-microbiome interactions [8]. In this study, we assessed fresh fecal specimens for concentrations of microbial- and host-generated metabolites.

## 2. Results

### 2.1. Fecal Metabolomics at Baseline and after Treatment with Different Fiber Sources

Fecal metabolite concentrations were compared at baseline and at the end of each 4-week feeding period for healthy and CKD cats. In total, 763 metabolites were detected across all test times. Of these, 33 fecal metabolites were different between healthy cats and CKD cats at baseline (after the 14-day pre-trial feeding period). Amongst these metabolites, 15 were increased and 18 were decreased in CKD cats compared with healthy cats.

Fecal metabolite concentrations were compared after feeding food A (scFOS) as a fiber source for 4 weeks: 48 metabolites differed from baseline in healthy cats and 64 metabolites differed from baseline in CKD cats. After feeding food B (apple pomace) as a fiber source for 4 weeks, 50 metabolites differed from baseline in healthy cats and 68 metabolites differed from baseline in CKD cats.

**Figure 1:**
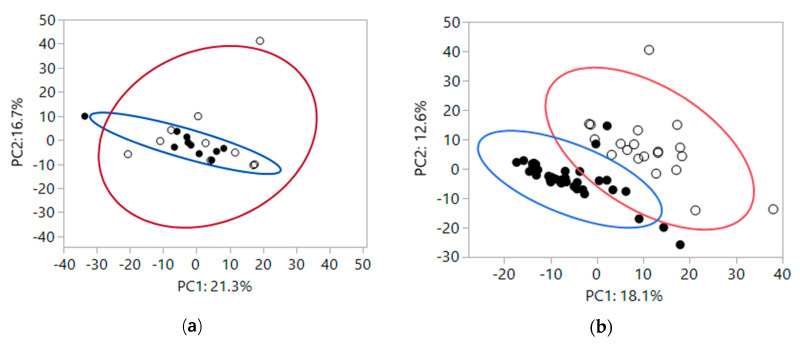
(**a**) At baseline fecal metabolites of healthy cats (closed circles) and chronic kidney disease (CKD) cats (open circles). Large colored circles represent 95% variance areas of healthy (blue) and CKD cats (red). The means of PC1 and PC2 were not significantly different (PC1, *P* = 0.25; PC2, *P* = 0.61). Variances were also not significantly different based on health status (PC1, *P* = 0.38; PC2, *P* = 0.10). (**b**) Fecal metabolites after feeding scFOS or apple pomace fiber sources for 4 weeks (closed circles) vs baseline (open circles). Large colored circles represent 95% variance areas at baseline (red) and after feeding fiber sources (blue). The means of PC1 and PC2 after feeding dietary fiber for four weeks were different (both *P* < 0.0001). Variances were not significantly different between baseline and after feeding fiber sources for 4 weeks (PC1, *P* = 0.75; PC2, *P* = 0.10).

To gain insight into how the fiber treatments impacted global fecal metabolomics data, a 2-dimensional principal component (PC) analysis was performed, which groups metabolites that track together and reduces the dimensions of the data down to two linear components. At baseline, fecal metabolites were not significantly different by health status (Figure 1a: PC1, *P* = 0.25; PC2, *P* = 0.61). Variance in fecal metabolites based on health was expressed by denoting the 95% variance areas with ellipses around healthy (blue circle) and CKD (red circle) cats. There was no significant difference in variance by health status (PC1, *P* = 0.38; PC2, *P* = 0.10). After feeding foods containing scFOS or apple pomace as sources of fiber, fecal metabolites clustered based on feeding dietary fiber, with baseline fecal metabolites being different from fecal metabolites present after feeding both fiber sources, shown in Figure 1b. The means of PC1 and PC2 for baseline and after feeding dietary fiber were different (mean ± SEM, PC1: 11.4 ± 2.1 and −5.8 ± 1.3, respectively and PC2: 6.9 ± 2.5 and −3.5 ± 1.1, respectively; both *P* < 0.0001). Table S1 includes all eigenvectors in PC1 and PC2. Variance in fecal metabolites based on fiber was expressed by denoting the 95% variance areas with ellipses at baseline (red circle) and after feeding fiber sources for 4 weeks (blue circle). Variances were not significantly different between baseline and after feeding fiber sources for 4 weeks (PC1, *P* = 0.75; PC2, *P* = 0.10). Some of the eigenvetor metabolites that changed the most after feeding fiber were increases in polyphenols such as glycitein, genistein sulfate, daidzein sulfate, and equol, and increases in products of starch digestion, such as maltose and maltotriose.

### 2.2. Treatment Differences in Fecal Metabolites Based on Health Status of Cats and Fiber Source Fed

For purposes of this report, we analyzed fecal metabolite concentrations of antioxidants; markers of oxidation and inflammation; methylated compounds; tricarboxylic acid (Citric or Krebs) cycle and urea cycle intermediates; other renal-associated metabolites; and primary and secondary bile acids. Fecal metabolite concentrations for antioxidants and markers of oxidation and inflammation are shown in Table 1. Across all time points of the study, based on overall effect of health (healthy cats versus CKD cats) there were few significant differences (gamma-CEHC and sphinganine concentrations were higher in healthy cats). All of the studied metabolites for tocopherol metabolism, and most for glutathione and sphingolipid metabolism in both healthy and CKD cats were affected by treatment with fiber (both scFOS and apple pomace). Compared with baseline, healthy cats had lower concentrations of alpha-tocopherol, delta-tocopherol, alpha-tocotrienol, gamma-tocotrienol, gamma-CEHC, and gamma-tocopherol/beta-tocopherol after consuming both fiber sources. In addition, healthy cats consuming apple pomace had lower concentrations of alpha-CEHC, albeit higher concentrations of alpha-CEHC sulfate. Healthy cats also had lower concentrations of sphinganine and sphingosine after consuming both fiber sources compared with baseline, and lower concentrations of sphingadienine after consuming scFOS. Compared with baseline, CKD cats had lower concentrations of delta-tocopherol, gamma-tocotrienol, gamma-tocopherol/beta-tocopherol, and cysteinylglycine after consuming both fiber sources. After consuming scFOS, CKD cats had higher concentrations of alpha-tocopherol acetate, but lower concentrations of alpha-tocotrienol and sphingadienine.

Comparing the two fiber sources, healthy cats had smaller decreases in alpha-tocopherol and alpha-tocopherol acetate after consuming scFOS compared with apple pomace, but greater decreases in alpha-tocotrienol and cysteinylglycine after consuming scFOS. CKD cats had greater decreases in alpha-tocotrienol, gamma-tocopherol/beta tocopherol, cysteinylglycine, and sphingadienine after consuming scFOS compared with apple pomace. There was an overall interaction for cysteinylglycine because healthy cats had no significant change from baseline concentrations, whereas CKD cats had significant decreases after consuming both fiber sources.

Across all time points of the study, for the metabolites related to methylation, based on overall effect of health (healthy cats versus CKD cats), glycine and betaine concentrations were higher in healthy cats. Glycine, dimethylglycine and betaine were affected by treatment (both scFOS and apple pomace; betaine was present in both treatments). Compared with baseline, healthy cats had increased concentrations of glycine, dimethylglycine and betaine after both treatments, whereas CKD cats had increases in dimethylglycine and betaine after treatment with apple pomace only. Comparing the two fiber sources, healthy cats had no differences between the two fiber sources, whereas CKD cats had a greater increase in betaine after consuming apple pomace compared with scFOS. There was an overall interaction for glycine because healthy cats had increased concentrations after consuming both fiber sources, but CKD cats had no change after consuming either fiber source.

Across all time points of the study, for some amino acid metabolites (valine, arginine and proline) and TCA and urea cycle intermediates, based on overall effect of health (healthy cats versus CKD cats) there were few significant differences (argininate and succinate concentrations were higher in healthy cats). Of the studied metabolites in the TCA cycle, none were affected by treatment with fiber (both scFOS and apple pomace). However, many of the urea cycle, arginine and the amino acid proline metabolites were affected by treatment with fiber (both scFOS and apple pomace). Compared with baseline, healthy cats had increased concentrations of arginine, norvaline, 2-oxoarginine, homocitrulline, dimethylarginine (SDMA+ADMA), N-acetylarginine, N-acetylcitrulline, and trans-4-hydroxyproline after treatment with both fiber sources, and increased concentrations of proline, N-monomethylarginine, and argininate after treatment with apple pomace only. Healthy cats had decreased concentrations of N-acetylproline after treatment with both fiber sources, and decreased concentrations of citrulline and N-methylproline after treatment with apple pomace fiber only. Compared with baseline, CKD cats had increased concentrations of alpha-ketoglutarate (with apple pomace fiber only) in the TCA cycle. For arginine, proline, and valine metabolites, CKD cats had increased concentrations of 2-oxoarginine and homocitrulline after treatment with both fiber sources, and increased norvaline after treatment with scFOS only. Compared with baseline, CKD cats had decreased concentrations of proline and N-acetylpropline after treatment with both fiber sources, and decreased N-alpha-acetylornithine after treatment with apple pomace only. Comparing the two fiber sources, healthy cats had a greater increase in N-monomethylarginine, and a greater decrease in N-methylproline concentrations with apple pomace compared with scFOS. There were no differences between the two fiber sources for CKD cats. There was an overall interaction for proline because healthy cats had a significant increase in concentration after treatment with apple pomace, whereas CKD cats had a significant decrease from baseline concentrations after consuming both fiber sources. In summary, in the TCA cycle, no changes in fecal metabolite concentrations were found after feeding both fiber sources. There were more effects with valine, arginine and proline metabolites, e.g., arginine, 2-oxoarginine, and homocitrulline; if concentrations were significantly different from baseline, they were increased. The dimethylarginines (SDMA and ADMA) and N-acetylarginine concentrations were increased from baseline after feeding both fiber sources in healthy cats, but not CKD cats.

Fecal metabolite concentrations for other renal-associated markers and metabolites are shown in Table 2. Across all time points of the study for creatine, tryptophan and benzoate metabolites, based on overall effect of health (healthy cats versus CKD cats) there were few significant differences (N-methylhydantoin, xanthurenate, and phenylpropionylglycine concentrations were higher in healthy cats; tryptamine concentrations were higher in CKD cats). Many of the studied metabolites were affected by treatment with fiber (both scFOS and apple pomace).

For creatine metabolites, compared with baseline, healthy cats had decreased concentrations of guanidinoacetate after consuming apple pomace, whereas CKD cats had decreased concentrations after consuming scFOS. Healthy cats had increased concentrations of creatinine after consuming apple pomace compared with baseline. CKD cats had decreased concentrations of N-methylhydantoin after consuming scFOS. Comparing fiber sources, there were no differences within healthy cats or within CKD cats for apple pomace and scFOS fiber sources, and no overall effect of interaction.

For tryptophan metabolites, compared with baseline, healthy cats had increased concentrations of tryptophan betaine and indolelactate after treatment with both fiber sources, and decreased concentrations of N-formylanthranilic acid, serotonin, and 2-aminophenol after treatment with both fiber sources. Feeding apple pomace to healthy cats caused increased concentrations of N-acetylkynurenine and indoleacetylglycine, and decreased concentrations of xanthurenate, tryptamine, and indole-3-carboxylic acid compared with baseline. Feeding scFOS to healthy cats caused increased picolinate concentrations. Compared with baseline, CKD cats had increased concentrations of tryptophan betaine and decreased concentrations of tryptophan, N-acetyltrytophan, N-formylanthranilic acid, 2-aminophenol, and valeryltryptophan after treatment with both fiber sources. Feeding apple pomace to CKD cats caused decreased concentrations of anthranilate, xanthurenate, serotonin, and indole. Feeding scFOS caused increased concentrations of 5-hydroxyindoleacetate and indoleacetate, and decreased concentrations of kynurenine, N-acetylkynurenine, tryptamine, and indolelactate. Comparing the two fiber sources, healthy and CKD cats had greater decreases in xanthurenate after consuming apple pomace compared with scFOS. Healthy cats also had a greater decrease in 5-hydroxyindoleaetate and smaller increase in picolinate concentrations after consuming apple pomace compared with scFOS. However, healthy cats had a greater increase in indoleacetylglycine concentrations after consuming apple pomace. There was an overall effect of interaction for tryptophan because CKD cats consuming both fiber sources had decreased concentrations whereas there was no change in concentrations for healthy cats. There was also an overall effect of interaction for indolelactate because healthy cats had increased concentrations for both fiber sources, whereas CKD cats had a significant decrease after consuming scFOS and no change in concentrations after consuming apple pomace.

For benzoate metabolites, compared with baseline, healthy cats had increased concentrations of hippurate, 2-hydroxyhippurate, 4-hydroxyhippurate, 4-hydroxybenzoate, and phenylpropionylglycine, and decreased concentrations of 3-(3-hydroyphenyl)propionate after treatment with both fiber sources. Feeding apple pomace to healthy cats caused increased concentrations of 4-methylcatechol sulfate, and decreased concentrations of 3-phenylpropionate (hydrocinnamate). Feeding scFOS to healthy cats caused increased concentrations of 2-(4-hydroxyphenyl)propionate. Compared with baseline, CKD cats had increased concentrations of 2-(4-hydroxyphenyl)propionate after treatment with both fiber sources. Feeding apple pomace caused increased concentrations of 4-methylcatechol sulfate and decreased concentrations of benzoate and p-cresol. Feeding scFOS to CKD cats caused decreased concentrations of 3-(3-hydroyphenyl)propionate sulfate and 3-(3-hydroyphenyl)propionate. Comparing the two fiber sources, both healthy and CKD cats had a greater increase in 4-methylcatechol sulfate after consuming apple pomace compared with scFOS. There was also an overall effect of interaction for 4-hydroxyhippurate perhaps due to the observation that healthy cats had increased concentrations after consuming both fiber sources and CKD cats had no change from baseline concentrations after feeding either fiber source. There was also an overall effect of interaction for 3-phenylpropionate (hydrocinnamate) because concentrations decreased after consuming apple pomace in healthy cats but did not change in CKD cats after feeding either fiber source.

Fecal metabolite concentrations for bile acid metabolites are shown in Table 3. At baseline, and across all time points of the study for the primary and secondary bile acid metabolites, based on overall effect of health (healthy cats versus CKD cats) there were no significant differences. Many of the studied metabolites were affected by treatment with fiber (both scFOS and apple pomace).

Compared with baseline, healthy cats had decreased concentrations of the primary bile acid beta-muricholate, and decreased concentrations of the secondary bile acids deoxycholate, isoursodeoxycholate, 7,12-diketolithocholate, and 7-ketodeoxycholate after treatment with both fiber sources. Only hyocholate, a secondary bile acid was increased after treatment with both fiber sources. Feeding apple pomace to healthy cats caused increased concentrations of the primary bile acids chenodeoxycholate and taurochenodeoxycholate, and increased concentrations of the secondary bile acids taurolithocholate 3-sulfate, tauroursodeoxycholate, taurocholenate sulfate, and taurochenodeoxycholate sulfate. Feeding apple pomace to healthy cats caused decreased concentrations of the secondary bile acids lithocholate, dehydrolithocholate, and 7alpha-hydroxycholestenone. Feeding scFOS to healthy cats caused decreased concentrations of the secondary bile acids deoxycholic acid sulfate, 12-dehydrocholate, and ursodeoxycholate sulfate. Feeding apple pomace to CKD cats caused increased concentrations of the primary bile acids chenodeoxycholate and taurochenodeoxycholate, and increased concentrations of the secondary bile acids tauroursodeoxycholate, hyocholate, and taurochenodeoxycholate sulfate. Feeding apple pomace to CKD cats caused decreased concentrations of the secondary bile acids deoxycholate. Feeding scFOS to CKD cats caused decreased concentrations of the primary bile acids cholate and cholate sulfate, and decreased concentrations of the secondary bile acids deoxycholic acid sulfate and ursodeoxycholate sulfate.

Comparing the two fiber sources, both healthy and CKD cats had a greater increase in the concentration of the primary bile acid taurochenodeoxycholate after consuming apple pomace compared with scFOS, and greater increases in the secondary bile acids tauroursodeoxycholate and hyocholate after consuming apple pomace compared with scFOS. In addition, healthy cats had greater increases in the secondary bile acids taurolithocholate 3-sulfate and taurocholenate sulfate after consuming apple pomace compared with scFOS. There was no overall effect of interaction for any of the bile acid metabolites.

## 3. Discussion

Kidney disease in cats [10] and human patients [12] is associated with altered concentrations of several metabolites in blood. For example, cats with CKD compared with healthy cats have higher blood concentrations of creatinine, urea, and some microbial and host tryptophan metabolites including several indole sulfates and kynurenate [10]. In human patients, fecal metabolites including tryptophan, free amino acids, and bile acids are altered with CKD [13,14]. Our goal was to compare the fecal metabolites of CKD and healthy cats after feeding two different fiber sources, knowing that the fecal microbiome in cats with CKD are more resistant to change compared with the fecal microbiome of healthy cats [10], similar to CKD and healthy humans [15]. In general, we have shown that changes in fecal metabolite concentrations did not routinely reflect plasma metabolite concentrations previously reported for these cats [10].

There were relatively few fecal metabolites (33 out of 763 metabolites detected, 4%) that differed between healthy and CKD cats at baseline. The PC analysis showed no significant difference in fecal metabolites based on health status at baseline. After feeding each fiber source, however, fecal metabolites were significantly different compared with baseline. Antiinflammatory polyphenols, including glycitein, genistein sulfate, daidzein sulfate and equol were among the fecal metabolites that increased from baseline concentrations after cats were fed foods containing either fiber treatment (Table S1). Increases in these metabolites is associated with reduced inflammation, endotoxemia and risk of developing cancer [16,17]. Thus, changes associated with feeding fiber were more significant than changes associated with disease status for fecal metabolites.

None-the-less there were some treatment differences in individual fecal metabolites based on health status of cats and fiber source fed, which are highlighted here. For organizational purposes, we divided metabolites into those that were generated by the host, e.g., those associated with oxidation, inflammation, methylation, and energy production, and those that could be generated either by the gut microbes or by the host, e.g., those associated with individual amino acids, urea, creatine, benzoate, and bile acid metabolism.

### 3.1. Fecal Metabololites Generated by the Host that Were Different after Treatment with Fiber Sources

With regard to markers of oxidation (tocopherol and glutathione metabolism), most of the fecal metabolite concentrations were decreased after consuming both fiber sources in healthy as well as CKD cats compared with baseline. Fecal excretion is a major route for elimination of vitamin E metabolites [18]. In CKD cats, fecal concentrations of gamma-tocopherol/beta-tocopherol and alpha-tocotrienol were lower after feeding scFOS compared with apple pomace, suggesting that even though both fibers helped decrease fecal losses, there was less lost in the feces after consuming scFOS compared with apple pomace. Previously, we reported that plasma concentrations of gamma-tocopherol/beta-tocopherol but not alpha-tocopherol were lower in CKD cats compared with healthy cats [10], although there was no effect of treatment with fiber in CKD cats on plasma vitamin E metabolite concentrations. Lower concentrations of gamma-tocopherol/beta-tocopherol and alpha-tocotrienol in feces of CKD cats fed scFOS might be because feeding this fiber to CKD cats resulted in greater microbial catabolism.

Similarly, CKD cats had a greater decrease in fecal cysteinylglycine concentrations (in the glutathione metabolism pathway) while consuming scFOS compared with apple pomace. Although, none of the glutathione metabolites in plasma [10] or feces were different based on health status at baseline, and feeding either fiber source did not affect plasma concentrations of glutathione metabolites [10]. Because the antioxidant glutathione conjugates with amino acids to form cysteinylglycine [19], reduced concentrations of cysteinylglycine in the feces of CKD cats consuming scFOS may suggest increased intestinal concentrations of glutathione, which is known to provide intestinal mucosal health benefits [20]. Similarly, there were lower fecal concentrations of 2-hydroxybutyric acids in CKD cats after consuming scFOS compared with apple pomace. Increased concentrations of 2-hydroxybutyric acids are associated with oxidative stress and impaired glucose regulation [21]. Previously we reported that CKD cats had significantly higher plasma concentrations of oxidized glutathione (GSSG) and cysteine-glutathione disulfide after feeding apple pomace compared with scFOS as a fiber source [10]. Higher plasma concentrations of GSSG and cysteine-glutathione disulfide along with higher fecal concentrations of 2-hydroxybutyric acids in CKD cats after feeding apple pomace compared with scFOS suggest that feeding apple pomace increases intestinal as well as systemic markers of oxidative stress.

We have shown previously that these two fiber sources had a significant differential effect on plasma sphingolipid metabolite concentrations of healthy and CKD cats [10], although there was no difference at baseline between the two groups. The increase in plasma sphingolipid metabolites noted in healthy cats after consuming scFOS, and the increase in CKD cats after consuming apple pomace [10] were not observed for fecal metabolite concentrations. Sphingolipids are building blocks of eukaryotic cell membrane and play a major role in inflammation [22,23]. Overall, CKD cats had lower fecal concentration of sphinganine compared with healthy cats. Sphingosine and sphingosine-1-phosphate are among the most studied mammalian sphingolipids. Sphingosine-1-phosphate is associated with chronic intestinal inflammation and cancer [22]. The accumulation of sphingosine, sphinganine or their 1-phosphate metabolites in blood or urine may cause renal toxicity [24,25,26]. The role that fecal concentrations of sphinganine play in kidney disease is not well understood. However, bacteria and viruses can use host sphingolipids to promote their virulence [27]. The reduction of fecal concentrations of sphingadienine in both healthy and CKD cats after feeding scFOS suggest a possible mechanism for the prebiotic in modulating the gut microbiota.

For the metabolites related to methylation, fecal concentrations were more likely to be increased for healthy cats (e.g., glycine, dimethylglycine, and betaine) than CKD cats (increased dimethylglycine and betaine after treatment with apple pomace only). Fecal concentrations of glycine and betaine were lower in CKD cats compared with healthy cats for both fiber treatments. Despite a similar concentration of betaine added to both test foods, fecal concentrations of betaine were higher only in CKD cats fed apple pomace, whereas in healthy cats, fecal concentrations were higher than baseline when fed either fiber source. Circulating concentrations of betaine were increased over baseline, but not statistically different by fiber source in CKD or healthy cats [10]. In the one carbon metabolism pathway, betaine donates a methyl group to convert the uremic toxin homocysteine to methionine and in the process becomes dimethylglycine [28]. Our studies showed that concentrations of dimethylglycine in feces and plasma of healthy and CKD cats [10] increased after feeding both fiber sources (not significant for CKD cats fed scFOS). Koistinen et al. [29] reported an increase in the abundance of beneficial gut bacteria including Bacteroides after mice were fed betaine-enriched food, which increased the concentrations of betainized metabolites in the colon. We previously showed that food containing scFOS significantly increased the abundance of an unclassified genus in the family S24-7, order Bacteroidales [10]. The betainized fecal metabolite, tryptophan betaine, was increased from baseline in the presence of both fiber sources in healthy and CKD cats. Further studies on microbial utilization of betaine may provide useful information on targeting the gut microbiome to balance the concentration of betaine and its metabolic end-products.

### 3.2. Fecal Metabololites Generated by the Gut Microbes or by the Host that Were Different after Treatment with Fiber Sources

Most gut microbiome–derived uremic solutes, as defined by Bush et al. [30], in the tryptophan and benzoate metabolism pathways, as well as bile acids, were not different between healthy and CKD cats at baseline. This may be because these uremic toxins play a more significant role in the later stages of kidney disease [31]. Interestingly, N-methylhydantoin concentrations were decreased overall in CKD cats compared with healthy cats. In CKD, circulating creatinine defuses into the intestine, whereupon the gut microbiota convert it to N-methylhydantoin. N-methylhydantoin is then reabsorbed into the blood and acts as a uremic toxin to the kidney tubular cells [32]. A lower concentration of N-methylhydantoin in feces of CKD cats may suggest increased uptake into the blood; however, our previous study did not show significant difference in blood concentrations between CKD and healthy cats [10], even though creatinine concentrations were higher in CKD cats [10]. Alternatively, lower concentrations might be because feeding this fiber to CKD cats resulted in greater microbial catabolism. Feeding scFOS fiber to CKD cats decreased fecal N-methylhydantoin concentrations from baseline, although there was no difference between the two fiber sources overall. Future studies on gut microbial utilization of creatinine in cats with CKD are necessary to understand the significance of decreased N-methylhydantoin fecal concentrations.

Urea, one of the traditional serum biomarkers for assessing glomerular filtration rate, was not detected in the fecal metabolome. Other serum biomarkers that increase with CKD include the dimethylarginines (SDMA and ADMA) and N-acetylarginine. Their concentrations were higher in plasma of CKD cats compared with healthy cats at all-time points [10], although in feces their concentrations increased from baseline after feeding both fiber sources in healthy cats, but not CKD cats. Citrulline, in the urea cycle, also had significantly higher concentrations in plasma of CKD cats [10], but concentrations were not different in the fecal metabolome. These findings indicate that fecal metabolite concentrations do not routinely reflect plasma metabolite concentrations.

In general, fecal metabolites for other renal-associated metabolites were different after feeding both fiber sources. The majority were decreased by fiber treatments. Both healthy and CKD cats had increased concentrations of tryptophan betaine, likely because of dietary inclusion of betaine. The CKD cats also had increased concentrations of indoleacetate and 5-hydroxyindoleacetate after consuming scFOS, and increased 4-methylcatechol sulfate and 2-(4-hydroxyphenyl)propionate after consuming apple pomace. However, both healthy and CKD cats had a greater increase in 4-methylcatechol sulfate after consuming apple pomace compared with scFOS.

Fecal concentrations of the uremic toxins xanthurenate, hippurate, 4-hydroxyhippurate, and 4-methylcatechol sulfate, as defined by Bush et al. [30] and Davies et al. [33], were significantly affected by fiber treatment, with xanthurenate decreasing with apple pomace treatment in both healthy and CKD cats, but hippurate, 4-hydroxyhippurate, and 4-methylcatechol sulfate increasing in healthy cats treated with apple pomace fiber. Concentration of 4-methylcatechol sulfate also increased in CKD cats treated with apple pomace fiber. We previously reported that plasma concentrations of several microbial uremic toxins in the benzoate metabolism pathway were altered by fiber treatment [10], more so in healthy cats treated with both fiber sources than in CKD cats who benefited more from treatment with scFOS. The uremic toxin 4-ethylphenylsulfate was increased in plasma of healthy cats treated with both fiber sources, and in plasma of CKD cats treated with apple pomace. Overall, these data suggest that scFOS may be a better fiber source for cats with CKD.

Increased fecal indole concentrations were reported previously in cats after consuming diets containing 4% FOS, and fecal acetate, propionate, and total short chain fatty acid concentrations increased in 4% pectin-supplemented cats [34]. We did not measure short chain fatty acid concentrations in our study, but did see an overall decrease in fecal indole concentrations in all cats fed both fiber sources. This is, likely because we fed lower concentrations of scFOS along with beta glucan, both of which changed tryptophan metabolism resulting in an overall decrease in fecal indole concentrations.

Fiber supplementation affected concentrations of both primary and secondary bile acids in the feces, regardless of health status. Although there were no significant differences in concentrations of fecal bile acids at baseline between healthy and CKD cats, both groups had higher fecal concentrations of the primary bile acid taurochenodeoxycholate after consumption of apple pomace compared with scFOS. Concentrations of many of the secondary bile acids, formed by dehydroxylation and deconjugation of primary bile acids by gut microbes, were altered after fiber treatment, with some significant differences based on type of fiber consumed. For example, both healthy and CKD cats had greater increases in the feces of the secondary bile acids tauroursodeoxycholate and hyocholate after consuming apple pomace compared with scFOS. In addition, healthy cats had greater increases in the secondary bile acids taurolithocholate 3-sulfate and taurocholenate sulfate after consuming apple pomace compared with scFOS. Secondary bile acids are potent farnesoid × receptor (FXR) agonists, and FXR agonists induce release of FRF-15 in the intestine and inhibition of the rate-limiting enzyme in bile acid synthesis, cholesterol 7α-hydroxylase in the liver (reviewed in Ridlon et al. [35]). Reduction in bile acid synthesis, coupled with downregulation of the apical sodium bile salt transporter in the ileum, leads to a less efficient enterohepatic circulation and results in a smaller, unconjugated hydrophobic bile acid pool in the blood. Hydrophilicity of the bile acid pool is associated with disease states. Although we did not report serum bile acids in these cats, it has been reported in humans that concentrations of serum total bile acids is increased in CKD patients [36,37], with a change in composition (increased secondary bile acids [37] and decreased percent secondary bile acids relative to total bile acids [14]). The bile of human CKD patients has been reported to have increased secondary bile acids and decreased primary bile acids composition [38]. Human studies of the fecal metabolome in early stages of CKD showed an increase in microbial genes related to secondary bile acid biosynthesis indicating that conversion of primary to secondary bile acids by intestinal microbes takes place in early renal function decline [39]. Wang et al. [40] showed that fecal and serum metabolomes of patients with end stage renal disease were correlated and characterized by accumulation of secondary bile acids. Thus, differential modulation of the microbiome-bile acid axis through dietary fiber may be of benefit in cats with CKD, although much remains to be learned.

## 4. Materials and Methods

All study protocols and this study were reviewed and approved by the Institutional Animal Care and Use Committee, Hill’s Pet Nutrition, Inc., Topeka, KS, USA (Permit Number: CP709), and complied with the National Institutes of Health Guide for the Care and Use of Laboratory Animals [41]. Cats were housed individually and allowed access to indoor runs. Cats also had exposure to natural light that varied with seasonal changes. All cats were provided with regular opportunities to exercise, with access to toys. Cats were owned by the commercial funders of this research or their affiliates, who gave permission for them to be included in this study. At the conclusion of the study, all cats were returned to the Hill’s Pet Nutrition, Inc. colony.

### 4.1. Participants and Study Design

This was a cross-over study within a split-plot design performed using healthy cats (n = 10) and CKD cats (IRIS stage 1, 2, and 3; n = 10). The study used a pre-trial food, Food A and Food B. The pre-trial food was a complete and balanced dry food designed to aid in the management of renal disease. Food A was the pre-trial food supplemented with betaine (0.500%), oat beta glucan (0.586%), and 0.407% scFOS. Food B was the pre-trial food supplemented with betaine (0.500%), oat beta glucan (0.586%), and 3.44% apple pomace. Cats were fed pre-trial food for 14-days and then were randomly assigned to Food A or Food B. Cats fed Food A for 4 weeks were then switched to Food B for 4 weeks. Cats fed Food B for 4 weeks were then switched to Food A for 4 weeks. All cats had access to electronic feeders whereby fresh food was offered daily with amounts available for consumption calculated to maintain body weight; water was available *ad libitum*. Actual daily food intake (g/day) was recorded for each cat.

All cats were of domestic shorthair breed. Demographic data is shown in Table 4. Cats in this study ranged in age from 4.8 to 9.3 years. Inclusion criteria were healthy cats or cats with CKD. Cats were determined to be healthy based on the results of an annual physical examination, complete blood count (CBC), serum biochemistries, and urinalysis. Cats were excluded from the healthy group if they were known to have problems eating new foods or problems with repeated blood sampling, and/or had any diagnosed disease condition such as inflammatory bowel disease, dermatitis, food allergy, cancer/tumor, liver disease, CKD, or chronic urinary tract infection. The criterion for removal from the study was development of any condition whereby removal would benefit the animal, including any cat refusing to eat, or inadequate food intake resulting in weight loss greater than 15% of body weight. No healthy cats were removed from the study.

Cats with CKD were determined to have IRIS stage 1 (n = 5), 2 (n = 4), and 3 (n = 1) CKD based on the results of an annual physical examination, CBC, serum biochemistries, and urinalysis. Cats with CKD included cats that were persistently azotemic with creatinine (Cr) 1.6 to 3.2 mg/dL over an extended period, typically for ≥3 months (n = 5); four of these cats had nephrolithiasis, including the cat with IRIS stage 3 CKD. Nonazotemic cats with kidney stones (n = 4) as well as one cat with abnormal kidneys found on physical examination and ultrasonographic imaging (n = 1; right kidney missing) were also included in the CKD group. Cats with any other abnormal clinical findings except CKD, IRIS stage 1, 2, and 3 were excluded from the CKD group, notwithstanding history and physical examination findings (changes in urine volume or changes in kidney size or shape) that were consistent with CKD. The criterion for removal of CKD cats from the study was the same as for healthy cats. The IRIS stage 3 CKD cat was removed from the study because of reduced food intake when he was switched to the apple pomace food; he only ate the pre-trial food and the scFOS food.

### 4.2. Foods

A complete and balanced dry food designed to aid in the management of renal disease was fed during a pre-trial period for 14 days (Table 5). Foods A and B were formulated similar to the food fed during the pre-trial-period, with the exception that beet pulp fiber in pre-trial food was reduced and treatment foods were supplemented with betaine (0.500%) and oat beta glucan (0.586%). In addition, Food A was supplemented with 0.407% scFOS fiber and Food B was supplemented with 3.44% apple pomace (Table 5). The source of beta glucan was oats, which contained 22% beta glucan (thus, 0.129% final concentration of beta glucan in the food); scFOS was 95% scFOS (thus, 0.387% final concentration of scFOS in the food) and therefore, 3× higher concentration than oat beta glucan. Apple pomace contained 43.2% total dietary fiber, 20.8 mg/g free polyphenol and 27.34 mg/g bound polyphenols. In addition, apple pomace contained 4.06% protein and 4.42% fat, as well as having 15.77% crude fiber, 12.03% soluble fiber, and 32.67% insoluble fiber on a dry matter basis.

The scFOS fed in this study is a well-defined fiber that is quickly fermented [42]. Apple pomace, on the other hand, is composed of fibers including pectins, cellulose, hemicelluloses, lignins and gums, and free and bound polyphenols, which are more slowly fermented in the hind gut [43]. It is a by-product of the apple juice industry. Crude fiber, which represents the type of dietary fiber that remains as a residue after food receives a standardized laboratory treatment with dilute acid and alkali, was similar for the two foods. (Fiber analysis methods are reviewed by deGoy et al. [44].) Such treatment dissolves all soluble fibers and some insoluble fibers. The residue, or crude fiber, is primarily composed of cellulose and lignin. Insoluble fiber, some of which can be fermented by colonic bacteria, adds bulk to stools and was 50% higher in the food containing apple pomace as the fiber source compared with food containing scFOS. Soluble fiber absorbs water and becomes a gel during the digestive process. Some soluble fiber is readily fermented in the colon generating gases and physiologically active by-products. Food containing apple pomace was 100% higher in soluble fiber compared with food containing scFOS.

All cat foods were prepared by Hill’s Pet Nutrition, Inc., and met the nutritional requirements for adult cats (≥1 year) as established by the Association of American Feed Control Officials (AAFCO). Food was available in dry form only. Macronutrient composition, and soluble and insoluble fiber concentrations of foods were determined by a commercial laboratory (Eurofins Scientific, Inc., Des Moines, IA). Proximate analyses were completed using the following techniques: moisture-AOAC 930.15; protein-AOAC 2001.11; fat-AOAC 954.02; fiber-AOAC 962.09; ash-AOAC 942.0; and soluble and insoluble fiber AOAC 991.43. Carbohydrate composition was determined by calculation. Food composition, expressed as percentage of food, as fed, is shown in Table 5. Vitamin, mineral, and fatty acid analyses were performed by the same commercial laboratory. Fatty acid (FA) concentrations were determined by gas chromatography of FA methyl esters, and were expressed as g/100 g of FAs as fed. The sum of dietary saturated FA (SFA) was determined as follows: 8:0+10:0+11:0+12:0+14:0+15:0+16:0+17:0+18:0+20:0+22:0+24:0. The sum of dietary monounsaturated FA (MUFA) was determined as follows: 14:1+15:1+16:1+17:1+18:1+20:1+22:1+24:1. The sum of dietary polyunsaturated FA (PUFA) was determined as follows: 18:2(n−6)+18:3(n−6)+18:3(n−3)+18:4(n−3)+20:2(n−6)+20:3(n−6)+20:3(n−3)+20:4(n−6)+20:4(n−3)+20:5(n−3)+21:5(n−3)+22:2(n−6)+22:4(n−6)+22:5(n−6)+22:5(n−3)+22:6(n−3).

All three foods contained similar concentrations (within analytical variance of targets) of protein, and had similar predicted caloric content. Foods A and B were otherwise similar with equal crude fiber (2.0 and 2.1% for Food A scFOS and Food B apple pomace, respectively) whereas the soluble fiber was 0.8 and 1.6%, respectively.

### 4.3. Fecal Sample Collection and Fecal Metabolome

Fecal samples were assessed at baseline (end of 14-day pre-trial period) and at the end of each 4-week feeding period to evaluate changes in fecal metabolites. Fecal samples were collected, homogenized, and frozen as aliquots within 1 h of defecation. Whole feces were homogenized thoroughly using Thinky Mixer model ARM-310 (THINKY USA, Inc., Laguna Hills, CA, USA). Homogenous samples were aliquoted into labeled cryovials. The tubes were snap-frozen immediately in liquid nitrogen followed by storing at −80 °C until further processing.

Analysis of fecal metabolomic profiles was performed by a commercial laboratory (Metabolon, Morrisville, NC, USA) as previously described [46]. Briefly, extracted supernatant was split and run on gas chromatography and liquid chromatography mass spectrometer platforms in randomized order. Gas chromatography (for hydrophobic molecules) and liquid chromatography (for hydrophilic molecules) were used to identify and provide relative quantification of small metabolites present in fecal samples. Endogenous biochemical included amino acids, peptides, carbohydrates, lipids, nucleotides, cofactors and vitamins. The complete fecal metabolome dataset is shown as a heat map of statistically significant biochemicals profiled in this study (Table S2).

### 4.4. Statistical Methods

Metabolomics data were log transformed before analyses were performed using ArraySudio (Omicsoft Corporation, Cary, NC, USA). A mixed model was used with cat identity as random effect to test whether means were different based on health status of cats (healthy versus CKD), type of fiber fed (scFOS versus apple pomace), or an interaction between health status and type of fiber fed. This test allows for unequal variances and has an approximate *t*-distribution with degrees of freedom estimated using Satterthwaite’s approximation. Significance was established when *P* ≤ 0.05 (for type 1 error) and *q* ≤ 0.1 (*q*-values were used to estimate false discovery rate in multiple comparisons). The mixed model was then used to test whether the difference of two paired observations from a single cat at baseline and the end of the 4 week feeding period was different than zero. This test was also used to test whether means were different after treatment with apple pomace versus scFOS as a fiber source within healthy cats and within CKD cats.

An independent *t*-test was used to test for differences of the means of the PC in the PC analysis of fecal metabolomics data at baseline comparing healthy and CKD cats. The mixed model was then used to test the means of PC1 and PC2 after treatment with fiber sources. Levene’s test was used to test whether the variance of PC1 and PC2 in the PC analysis were different.

## 5. Conclusions

There were relatively few fecal metabolites that differed between healthy and CKD cats at baseline. Feeding both sources of fiber caused the fecal metabolites to change from baseline, such that fiber had a larger impact than disease status. Changes in fecal metabolite concentrations did not routinely reflect plasma metabolite concentrations previously reported for these cats. Be that as it may, the results of this study on the fecal metabolome in healthy and CKD cats after feeding two different fiber sources does not change our previous conclusion that health status impacts the influence of dietary fermentable fibers on the feline plasma metabolome and fecal microbiome [10]. There are just lesser effects on the fecal metabolome. We believe that a more readily fermented fiber such as scFOS is preferable to apple pomace as a fiber source for cats with CKD.

Nonetheless, there were some treatment differences in individual fecal metabolites based on health status of cats and type of fiber treatment. Perhaps most interesting was the effect of dietary fibers on concentrations of fecal uremic toxins (many decreased, e.g., xanthurenate; although in healthy cats some increased more when feeding apple pomace compared with scFOS, e.g., hippurate, 4-hydroxyhippurate, and 4-methylcatechol sulfate; the latter was also increased in CKD cats). Likewise, changes in secondary bile acid concentrations were more numerous in healthy cats compared with CKD cats. Cats in both groups had greater increases in some secondary bile acids after consuming apple pomace compared with scFOS, e.g., tauroursodeoxycholate and hyocholate. Knowing that secondary bile acids are potent FXR agonists, future studies looking at the hydrophilicity and size of the blood bile acid pool associated with healthy and CKD cats and type of fiber fed would be interesting.

## Figures and Tables

**Table 1:** Relative fecal metabolite concentrations for antioxidants, markers of oxidation and inflammation, methylated metabolite, TCA cycle, and urea cycle intermediates in healthy cats (H cats) and chronic kidney disease (CKD) cats at baseline (after feeding pretrial food ^1^ for 14 days) and after feeding food A ^2^ (containing scFOS) or food B ^3^ (containing apple pomace) as fiber sources for 4 weeks each.

	Mean Values ^*^						Group Effect ^‡^				
	H Cats			CKD Cats			Overall Effect of Health	Overall Effect of Both Treatments	H Cats: Effect of Treatment with Food B versus Food A ^¥^	CKD Cats: Effect of Treatment with Food B versus Food A ^¥^	Overall Effect of Interaction
Metabolites	BSL ^#^	Food A	Food B	BSL ^#^	Food A	Food B	*P*-Value	*P*-Value	*P*-Value	*P*-Value	*P*-Value
**Tocopherol Metabolism**											
alpha-tocopherol	1.11	1.00 ^§^	0.94 ^§^	1.03	0.99	0.93 ^§^	0.39	0.000	0.02	0.08	0.06
alpha-tocopherol acetate	0.94	1.09	0.89	0.83	1.02 ^§^	1.01	0.86	0.02	0.01	0.85	0.14
delta-tocopherol	1.31	0.97 ^§^	0.88 ^§^	1.23	0.96 ^§^	0.94 ^§^	0.91	0.000	0.02	0.74	0.09
alpha-tocotrienol	1.21	0.85 ^§^	1.02 ^§^	1.20	0.90 ^§^	1.07	0.56	0.000	0.003	0.000	0.26
gamma-tocotrienol	1.37	0.79 ^§^	0.85 ^§^	1.37	0.90 ^§^	1.00 ^§^	0.30	0.000	0.42	0.02	0.16
gamma-CEHC	1.51	1.20 ^§^	1.04 ^§^	0.93	0.67	0.68	0.04	0.01	0.69	0.78	0.66
alpha-CEHC sulfate	0.94	1.04	1.33 ^§^	0.63	0.64	0.88	0.15	0.03	0.13	0.66	0.68
alpha-CEHC	1.52	1.45	0.96 ^§^	0.97	0.83	0.59	0.10	0.01	0.02	0.23	0.71
gamma-tocopherol/beta-tocopherol	1.80	0.90 ^§^	0.97 ^§^	1.67	0.88 ^§^	1.02 ^§^	0.62	0.000	0.04	0.000	0.02
**Glutathione Metabolism**											
cysteinylglycine	0.94	0.78	0.99	1.29	0.75 ^§^	1.00 ^§^	0.61	0.000	0.02	0.01	0.01
5-oxoproline	1.59	1.80	1.61	1.49	1.19	1.79	0.30	0.53	0.63	0.22	0.43
2-hydroxybutyrate/2-hydroxyhydroxyisobutyrate	0.86	1.25	1.38	0.86	0.93	1.85	0.66	0.04	0.91	0.04	0.31
**Sphingolipid Metabolism**											
sphinganine	1.85	1.09 ^§^	1.04 ^§^	1.09	1.01	0.80	0.04	0.000	0.82	0.49	0.02
sphingosine	1.52	0.99 ^§^	0.96 ^§^	1.14	1.06	0.90	0.47	0.002	0.81	0.65	0.10
sphingadienine	1.11	0.93 ^§^	1.05	1.08	0.94 ^§^	1.07	0.94	0.000	0.03	0.004	0.57
**Glycine, Serine, and Threonine Metabolism**											
glycine	0.90	1.20 ^§^	1.49 ^§^	0.84	0.78	0.85	0.01	0.003	0.08	0.36	0.01
sarcosine	0.96	1.03	1.20	0.85	0.77	1.02	0.18	0.53	0.49	0.59	0.96
dimethylglycine	1.06	1.38 ^§^	1.36 ^§^	0.80	1.11	1.49 ^§^	0.27	0.01	0.99	0.57	0.81
betaine	0.93	1.82 ^§^	2.56 ^§^	0.58	0.64	1.20 ^§^	0.003	0.000	0.14	0.01	0.08
TCA Cycle											
citrate	0.90	1.24	1.63	0.94	0.89	1.01	0.18	0.43	0.76	0.46	0.52
alpha-ketoglutarate	0.91	1.79	1.19	0.98	1.04	1.69 ^§^	0.99	0.08	0.50	0.09	0.23
succinate	1.28	1.14	1.54	1.11	0.83	0.89	0.01	0.48	0.22	0.70	0.41
fumarate	1.49	1.00	0.89	1.54	1.25	1.35	0.72	0.27	0.75	0.56	0.44
malate	1.30	1.32	1.58	0.89	1.22	1.25	0.23	0.13	0.28	0.78	0.83
**Urea Cycle**											
arginine	0.53	1.46 ^§^	1.35 ^§^	0.59	0.85	0.87	0.06	0.001	0.75	0.67	0.02
argininosuccinate	0.10	0.55	0.33	0.11	0.45	0.52	0.97	0.13	0.98	0.92	0.95
ornithine	1.21	1.21	1.06	1.08	1.11	0.99	0.23	0.65	0.42	0.74	0.78
citrulline	1.14	0.98	0.93 ^§^	1.07	1.03	0.93	0.98	0.05	0.68	0.29	0.62
**Valine, Arginine, and Proline Metabolism**											
norvaline	0.72	2.34 ^§^	2.57 ^§^	0.79	1.44 ^§^	1.26	0.11	0.000	0.86	0.35	0.09
2-oxoarginine	0.64	1.68 ^§^	1.32 ^§^	0.59	1.09 ^§^	1.45 ^§^	0.23	0.000	0.46	0.21	0.18
homocitrulline	0.85	1.11 ^§^	1.04 ^§^	0.98	1.59 ^§^	1.34 ^§^	0.42	0.000	0.79	0.99	0.15
proline	1.05	1.23	1.37 ^§^	1.35	0.88 ^§^	1.12 ^§^	0.46	0.06	0.33	0.12	0.001
dimethylarginine (SDMA+ADMA)	0.81	1.12 ^§^	1.11 ^§^	0.99	1.06	1.11	0.89	0.005	0.98	0.66	0.03
N-acetylarginine	0.80	1.24 ^§^	1.14 ^§^	0.86	1.04	0.98	0.34	0.02	0.64	0.55	0.20
N-acetylcitrulline	0.77	1.21 ^§^	1.15 ^§^	0.85	1.03	0.93	0.30	0.01	0.64	0.50	0.42
N-acetylproline	1.60	0.82 ^§^	0.82 ^§^	1.62	0.76 ^§^	0.71 ^§^	0.59	0.000	0.94	0.72	0.50
N-delta-acetylornithine	1.11	1.01	0.96	1.06	0.88	1.20	0.42	0.61	0.86	0.37	0.49
N-alpha-acetylornithine	1.24	1.17	0.98	1.14	0.93	0.85 ^§^	0.10	0.01	0.17	0.51	0.68
N-methylhydroxyproline	0.86	0.86	0.89	0.99	0.88	1.07	0.33	0.40	0.50	0.23	0.91
trans-4-hydroxyproline	0.75	1.31 ^§^	1.17 ^§^	0.98	0.99	1.08	0.32	0.10	0.58	0.66	0.03
N-methylproline	1.70	1.60	0.63 ^§^	1.68	1.47	0.75	0.94	0.002	0.001	0.16	0.38
N-monomethylarginine	0.82	0.78	1.17 ^§^	1.08	0.90	1.11	0.44	0.02	0.002	0.41	0.07
argininate	1.05	1.33	1.70 ^§^	0.86	0.82	0.93	0.01	0.20	0.18	0.59	0.11

^1^ Baseline (BSL; pre-trial) food was a complete and balanced dry food designed to aid in the management of renal disease. ^2^ Food A was prepared by Hill’s Pet Nutrition, Inc. and was similar to the pre-trial food, with the exception that it was supplemented with betaine (0.500%), oat beta glucan (0.586%), and 0.407% short chain FOS (scFOS). ^3^ Food B was prepared by Hill’s Pet Nutrition, Inc. and was similar to the pre-trial food, with the exception that it contained 0.500% betaine, 0.586% oat beta glucan, and 3.44% apple pomace. * For each metabolite, mean value is the group mean of re-scaled data to have median equal to 1. ^‡^ A mixed model was used with cat identity as random effect to test whether means were different based on health status of cats (healthy versus CKD), type of fiber fed (scFOS versus apple pomace), or an interaction between health status and type of fiber fed. ^¥^ The mixed model was used to test whether means were different after treatment with apple pomace versus scFOS as a fiber source within healthy cats and within CKD cats. ^#^ The mixed model was used to test whether means were different at baseline based on health status of cats (healthy versus CKD). There were no differences. ^§^ The mixed model was used to test whether the difference of two paired observations from a single cat at baseline and the end of the 4 week feeding period was different than zero. Significance was established when *P* ≤ 0.05 (for type 1 error) and *q* ≤ 0.1 (*q*-values were used to estimate false discovery rate in multiple comparisons.

**Table 2:** Relative fecal metabolite concentrations for other renal-associated markers and metabolites in healthy cats (H cats) and CKD cats at baseline (after feeding pretrial food ^1^ for 14 days) and after feeding food A ^2^ (containing scFOS) or food B ^3^ (containing apple pomace) as fiber sources for 4 weeks each.

	Mean Values ^*^						Group Effect ^‡^				
	H Cats			CKD Cats			Overall Effect of Health	Overall Effect of Both Treatments	H Cats: Effect of Treatment with Food B versus Food A ^¥^	CKD Cats: Effect of Treatment with Food B versus Food A ^¥^	Overall Effect of Interaction
Metabolites	BSL ^#^	Food A	Food B	BSL ^#^	Food A	Food B	*P*-Value	*P*-Value	*P*-Value	*P*-Value	*P*-Value
**Creatine Metabolism**											
guanidinoacetate	1.83	1.07	0.84 ^§^	1.49	1.05 ^§^	1.43	0.84	0.02	0.46	0.28	0.42
creatine	0.98	1.16	1.54	1.42	0.76	1.45	0.26	0.20	0.37	0.33	0.78
creatinine	0.92	1.04	1.40 ^§^	1.10	0.72	1.38	0.26	0.16	0.24	0.19	0.38
N-methylhydantoin	1.00	1.24	1.53	0.88	0.26 ^§^	0.58	0.02	0.27	0.53	0.17	0.10
**Tryptophan Metabolism**											
tryptophan	1.19	1.57	1.61	1.64	0.85 ^§^	0.83 ^§^	0.14	0.15	0.98	0.65	0.005
N-acetyltryptophan	1.25	1.21	1.24	1.67	0.85 ^§^	0.84 ^§^	0.36	0.000	0.79	0.76	0.02
tryptophan betaine	0.55	1.01 ^§^	1.03 ^§^	0.55	0.93 ^§^	0.92 ^§^	0.19	0.000	0.61	0.67	0.31
kynurenine	1.02	0.96	0.97	1.27	0.95 ^§^	1.10	0.74	0.02	0.96	0.19	0.17
N-acetylkynurenine (2)	0.79	0.96	1.38 ^§^	1.83	1.32 ^§^	1.34	0.36	0.07	0.08	0.12	0.02
kynurenate	1.03	0.95	0.92	1.20	1.07	1.30	0.30	0.26	0.59	0.06	0.22
N-formylanthranilic acid	1.59	1.04 ^§^	0.86 ^§^	1.61	1.32 ^§^	0.89 ^§^	0.81	0.000	0.15	0.14	0.81
anthranilate	1.12	0.82	0.74	1.45	0.75	0.70 ^§^	0.48	0.03	0.70	0.58	0.96
xanthurenate	1.25	1.22	0.88 ^§^	0.99	0.96	0.79 ^§^	0.05	0.000	0.001	0.03	0.61
picolinate	0.90	1.25 ^§^	0.98	1.10	1.08	0.96	0.87	0.08	0.04	0.62	0.10
serotonin	1.31	0.95 ^§^	0.83 ^§^	1.19	1.15	0.90 ^§^	0.65	0.000	0.14	0.07	0.02
5-hydroxyindoleacetate	1.00	1.09	0.83	1.05	1.18 ^§^	0.90	0.59	0.02	0.01	0.14	0.25
tryptamine	1.85	0.93	0.76 ^§^	9.41	3.04 ^§^	6.76	0.05	0.001	0.41	0.08	0.15
indolelactate	0.87	2.28 ^§^	3.02 ^§^	1.28	0.79 ^§^	1.05	0.06	0.22	0.39	0.32	0.002
indoleacetate	1.17	0.85	0.89	0.69	0.93 ^§^	0.99	0.68	0.58	0.70	1.00	0.07
indolepropionate	1.77	1.94	1.77	2.57	0.85	0.85	0.70	0.05	0.18	0.77	0.74
indole	5.74	1.01	1.22	6.33	3.59	1.05 ^§^	0.46	0.05	0.91	0.42	0.45
indole-3-carboxylic acid	1.18	0.83	0.56 ^§^	0.95	1.06	0.81	0.53	0.24	0.39	0.83	0.09
indoleacetylglycine	0.99	1.15	2.34 ^§^	1.16	1.19	1.72	0.35	0.01	0.04	0.10	0.13
2-aminophenol	2.38	0.93 ^§^	0.93 ^§^	2.62	0.86 ^§^	0.93 ^§^	0.62	0.000	0.96	0.89	0.14
valeryltryptophan	1.60	1.34	1.19	2.06	0.62 ^§^	0.60 ^§^	0.25	0.000	0.49	0.99	0.19
**Benzoate Metabolism**											
hippurate	0.32	0.90 ^§^	1.85 ^§^	0.57	0.67	1.50	0.29	0.000	0.03	0.39	0.06
2-hydroxyhippurate (salicylurate)	0.67	1.71 ^§^	2.07 ^§^	0.75	1.11	1.16	0.12	0.001	0.50	0.99	0.03
4-hydroxyhippurate	0.84	1.39 ^§^	2.27 ^§^	1.23	1.11	0.98	0.09	0.02	0.03	0.78	0.01
benzoate	1.03	0.98	1.00	1.04	0.89	0.88 ^§^	0.25	0.12	0.79	0.43	0.29
4-hydroxybenzoate	1.05	1.49 ^§^	1.37 ^§^	2.00	1.18	1.40	0.86	0.40	0.94	0.58	0.08
catechol sulfate	1.20	0.93	1.25	0.86	0.81	1.12	0.28	0.26	0.35	0.57	0.81
4-methylcatechol sulfate	1.70	1.33	3.94 ^§^	1.36	0.80	2.93 ^§^	0.23	0.000	0.000	0.004	0.61
p-cresol	1.12	1.07	1.01	1.08	0.94	0.76 ^§^	0.31	0.03	0.42	0.25	0.66
p-cresol sulfate	1.31	0.84	1.17	0.87	0.75	0.79	0.24	0.71	0.20	0.92	0.60
phenylpropionylglycine	0.63	1.61 ^§^	2.99 ^§^	0.43	0.43	0.51	0.01	0.01	0.20	0.85	0.03
3-(3-hydroxyphenyl) propionate sulfate	1.34	0.85	0.96	0.93	0.56 ^§^	0.73	0.33	0.09	0.52	0.76	0.63
2-(4-hydroxyphenyl) propionate	0.45	0.80 ^§^	0.61	0.45	1.19 ^§^	0.83 ^§^	0.43	0.001	0.22	0.33	0.74
3-(3-hydroxyphenyl) propionate	1.85	0.90 ^§^	0.75 ^§^	1.93	0.78 ^§^	0.86	0.60	0.000	0.67	0.62	0.56
3-(4-hydroxyphenyl) propionate	1.29	1.04	1.07	1.10	1.02	1.22	0.64	0.27	0.96	0.16	0.61
3-phenylpropionate (hydrocinnamate)	9.21	5.70	2.57 ^§^	7.63	16.56	11.69	0.83	0.14	0.09	0.37	0.01

^1–3, *, ‡, ¥, #, §^ See Table 1.

**Table 3:** Relative fecal metabolite concentrations for primary and secondary bile acid metabolites in healthy cats (H cats) and CKD cats at baseline (after feeding pretrial food ^1^ for 14 days) and after feeding food A ^2^ (containing scFOS) or food B ^3^ (containing apple pomace) as fiber sources for 4 weeks each.

	Mean Values ^*^						Group Effect ^‡^				
	H Cats			CKD Cats			Overall Effect of Health	Overall Effect of Both Treatments	H Cats: Effect of Treatment with Food B versus Food A ^¥^	CKD Cats: Effect of Treatment with Food B versus Food A ^¥^	Overall Effect of Interaction
Metabolites	BSL ^#^	Food A	Food B	BSL ^#^	Food A	Food B	*P*-Value	*P*-Value	*P*-Value	*P*-Value	*P*-Value
**Primary Bile Acid Metabolism**											
cholate	1.41	1.10	1.20	1.41	0.94 ^§^	0.96	0.15	0.11	0.71	0.56	0.70
glycocholate	0.70	1.09	1.06	0.54	0.38	0.24	0.06	0.82	0.59	0.35	0.11
taurocholate	1.25	1.69	1.90	1.78	1.51	2.20	0.92	0.29	0.10	0.55	0.54
chenodeoxycholate	0.68	0.77	1.14 ^§^	0.74	0.74	1.11 ^§^	0.96	0.001	0.03	0.09	0.74
taurochenodeoxycholate	0.22	0.51	1.66 ^§^	0.61	0.68	2.50 ^§^	0.67	0.000	0.001	0.01	0.74
beta-muricholate	2.44	0.93 ^§^	0.79 ^§^	2.20	1.43	0.82	0.88	0.07	0.87	0.32	0.24
cholate sulfate	1.24	0.97	1.17	2.52	1.01 ^§^	1.16	0.35	0.05	0.64	0.15	0.12
**Secondary Bile Acid Metabolism**											
deoxycholate	2.41	1.23 ^§^	0.87 ^§^	1.48	1.16	0.71 ^§^	0.52	0.000	0.05	0.12	0.59
deoxycholic acid sulfate	0.95	0.17 ^§^	0.43	0.82	0.11 ^§^	0.31	0.91	0.01	0.34	0.46	0.99
taurodeoxycholate	1.38	1.26	0.97	0.82	0.88	0.89	0.58	0.40	0.75	0.81	0.86
lithocholate	2.98	1.54	1.24 ^§^	2.19	2.15	1.39	0.88	0.13	0.32	0.76	0.33
taurolithocholate 3-sulfate	0.73	0.81	2.23 ^§^	2.51	1.36	2.73	0.61	0.003	0.01	0.05	0.62
ursodeoxycholate	1.10	0.87	1.02	1.27	1.35	1.19	0.49	0.55	0.13	0.91	0.25
isoursodeoxycholate	1.49	0.94 ^§^	0.78 ^§^	1.20	1.13	0.99	0.72	0.01	0.33	0.68	0.08
tauroursodeoxycholate	0.39	0.66	1.30 ^§^	0.51	0.55	1.33 ^§^	0.92	0.000	0.01	0.01	0.89
dehydrolithocholate	3.89	8.28	2.77 ^§^	2.97	1.51	4.38	0.77	0.62	0.23	0.26	0.10
7,12-diketolithocholate	3.64	3.52 ^§^	1.14 ^§^	1.47	0.65	3.70	0.93	0.03	0.72	0.16	0.27
7-ketolithocholate	1.68	0.86	1.09	1.18	1.30	2.16	0.32	0.46	0.33	0.43	0.99
hyocholate	0.68	0.94 ^§^	1.63 ^§^	0.56	0.73	1.69 ^§^	0.20	0.000	0.000	0.000	0.46
3-dehydrocholate	2.18	1.33	1.28	1.26	0.86	1.33	0.19	0.14	0.90	0.46	0.65
12-dehydrocholate	2.51	1.13 ^§^	1.42	1.22	0.92	1.29	0.07	0.02	0.60	0.20	0.76
taurocholenate sulfate	0.67	0.82	2.13 ^§^	1.54	0.82	2.02	0.77	0.002	0.01	0.03	0.40
7-ketodeoxycholate	2.20	1.01 ^§^	0.94 ^§^	1.45	0.99	1.67	0.51	0.01	0.95	0.17	0.38
7alpha-hydroxycholestenone	1.12	1.02	0.90 ^§^	1.07	1.05	1.04	0.56	0.03	0.03	0.88	0.08
3b-hydroxy-5-cholenoic acid	1.45	1.11	1.06	1.03	1.20	1.09	0.67	0.84	0.60	0.99	0.12
taurochenodeoxycholate sulfate	0.63	0.68	1.51 ^§^	0.55	1.03	1.54 ^§^	0.75	0.01	0.14	0.40	0.89
ursodeoxycholate sulfate (1)	3.37	0.77 ^§^	1.99	4.00	0.66 ^§^	1.91	0.71	0.01	0.69	0.07	0.58
ursocholate	1.35	1.09	1.16	1.98	1.11	1.33	0.39	0.11	0.68	0.46	0.89

^1–3, *, ‡, ¥, #, §^ See Table 1.

**Table 4:** Demographic data, mean (standard deviation), at baseline for healthy adult cats (n = 10) and cats with IRIS stage 1, 2, and 3 chronic kidney disease (CKD; n = 10).

Cats	Healthy	CKD
Age, years	7.2 (1.3)	8.0 (1.8)
Sex	8 spayed females, 2 neutered males	5 spayed females, 5 neutered males
Body weight, kg	4.55 (0.68)	5.40 (0.85)

**Table 5:** Composition of pre-trial food ^1^, Food A ^2^, and Food B ^3^.

Nutrient	Pre-Trial Food	Food A with scFOS	Food B with Apple Pomace
Moisture	5.47	5.34	5.97
Protein	27.6	28.0	27.3
Fat	19.9	19.5	19.5
Atwater Energy, ^4^ kcal/kg	4101	4063	4032
Ash	4.50	4.42	4.59
Crude Fiber	1.3	2.0	2.1
Insoluble Fiber	4.0	3.4	5.1
Soluble Fiber	1.3	0.8	1.6
Total Dietary Fiber	5.3	4.2	6.7
Calcium	0.73	0.72	0.79
Phosphorus	0.64	0.51	0.47
Sodium	0.24	0.22	0.22
ARA [20:4 (n−6)]	0.04	0.04	0.04
EPA [20:5 (n−3)]	<0.01	0.02	0.02
DHA [22:6 (n−3)]	0.01	0.03	0.02
SFA ^5^	6.32	6.59	6.72
MUFA ^6^	7.40	8.04	8.24
PUFA ^7^	9.58	7.83	8.25
Total FA	18.56	18.61	19.16
(n−6) FA ^8^	4.10	3.70	3.90
(n−3) FA ^9^	0.69	0.22	0.23
(n−6):(n−3) ratio	5.9	16.8	17.0

^1^ Pre-trial food was a complete and balanced dry food designed to aid in the management of renal disease. All analytical values are expressed as percentage of food, as fed, unless otherwise indicated. ^2^ Food A was prepared by Hill’s Pet Nutrition, Inc. and was similar to the pre-trial food, with the exception that it was supplemented with betaine (0.500%), oat beta glucan (0.586%), and 0.407% scFOS. ^3^ Food B was prepared by Hill’s Pet Nutrition, Inc. and was similar to the pre-trial food, with the exception that it contained 0.500% betaine, 0.586% oat beta glucan, and 3.44% apple pomace. ^4^ Energy calculated using the modified Atwater factors as described [45]. ^5^ Sum of the SFA: 8:0+10:0+11:0+12:0+14:0+15:0+16:0+17:0+18:0+20:0+22:0+24:0. ^6^ Sum of the MUFA: 14:1+15:1+16:1+17:1+18:1+20:1+22:1+24:1. ^7^ Sum of the PUFA: 18:2(n−6)+18:3(n−6)+18:3(n−3)+18:4(n−3)+20:2(n−6)+20:3(n−6)+20:3(n−3)+20:4(n−6)+20:4(n−3)+20:5(n−3)+21:5(n−3)+22:2(n−6)+22:4(n−6)+22:5(n−6)+22:5(n−3)+22:6(n−3). ^8^ Sum of the (n−6) fatty acids. ^9^ Sum of the (n−3) fatty acids.

## Supplementary Materials

The following are available online at https://www.mdpi.com/2218-1989/10/7/281/s1, Table S1: Eigenvector metabolites that contributed to separation of the two groups in Figure 1b, PC1 and PC2. Table S2: The complete dataset for feline fecal metabolites is shown as a heat map of statistically significant biochemicals profiled in this study.
