# Supplementary material for: Changes in the Fecal Metabolome Are Associated with Feeding Fiber Not Health Status in Cats with Chronic Kidney Disease
Source: Metabolites. 2020 Jul 9;10(7):281. doi: 10.3390/metabo10070281 (PMC7407581; doi:10.3390/metabo10070281)
Supplement: Supplementary file 1 [file metabolites-10-00281-s001.zip › Table S1 Eigenvectors for Figure 1b May 29 2020.docx]

**Table S1.** Eigenvector metabolites that contributed to separation of the two groups in Figure 1(b), PC1 and PC2.

| **Fecal metabolites** | **PC1** | **Fecal metabolites** | **PC2** |
| --- | --- | --- | --- |
| 5-methylthioadenosine (MTA) | -0.07375 | homocitrulline | -0.07096 |
| N-acetylglycine | -0.0694 | equol | -0.06458 |
| orotate | -0.06741 | 3-hydroxysuberate | -0.06333 |
| glycitein | -0.06661 | 3beta-hydroxy-5-cholestenoate | -0.05564 |
| NAD+ | -0.06568 | arachidoyl ethanolamide (20:0) | -0.05533 |
| 5-(galactosylhydroxy)-L-lysine | -0.0652 | N-acetyl-3-methylhistidine | -0.05527 |
| 1-palmitoyl-GPC (16:0) | -0.06519 | 7-hydroxycholesterol (alpha or beta) | -0.05523 |
| 1-palmitoyl-galactosylglycerol (16:0) | -0.06378 | glycosyl ceramide (d18:1/20:0, d16:1/22:0) | -0.05504 |
| arginine | -0.06323 | 3b-hydroxy-5-cholenoic acid | -0.055 |
| 3-hydroxyadipate | -0.06289 | behenoyl ethanolamide (22:0) | -0.05446 |
| maltose | -0.06226 | 5-hydroxyindoleacetate | -0.05365 |
| maltotriose | -0.06203 | biotin | -0.05328 |
| choline | -0.06198 | glycosyl-N-stearoyl-sphingosine (d18:1/18:0) | -0.05125 |
| genistein sulfate | -0.06178 | 1-methylurate | -0.05092 |
| alpha-hydroxycaproate | -0.0597 | N-carbamoylalanine | -0.05023 |
| 1-stearoyl-2-arachidonoyl-GPE (18:0/20:4) | -0.05901 | lignoceroylcarnitine (C24) | -0.04832 |
| daidzein sulfate (2) | -0.05884 | lignoceroyl ethanolamide (24:0) | -0.04811 |
| gamma-glutamylisoleucine | -0.05845 | behenoylcarnitine (C22) | -0.04736 |
| AMP | -0.05811 | 21-hydroxypregnanolone monosulfate (1) | -0.04723 |
| cytosine | -0.0579 | 2-hydroxymyristate | -0.04704 |
| arachidonoyl ethanolamide | -0.0573 | tryptophan betaine | -0.0469 |
| caproate (6:0) | -0.05687 | nervonoyl ethanolamide (24:1) | -0.04644 |
| palmitoleoyl ethanolamide | -0.05677 | hydroxystearate sulfate | -0.04485 |
| daidzein | -0.05591 | glycosyl-N-palmitoyl-sphingosine (d18:1/16:0) | -0.04471 |
| alpha-ketoglutaramate | -0.05581 | 3beta,7alpha-dihydroxy-5-cholestenoate | -0.04457 |
| 1-stearoyl-2-arachidonoyl-GPC (18:0/20:4) | -0.05576 | mead acid (20:3n9) | -0.04446 |
| tryptophan betaine | -0.05543 | carotene diol (2) | -0.04443 |
| sphingomyelin (d18:1/20:0, d16:1/22:0) | -0.05527 | ximenoyl ethanolamide (26:1) | -0.0434 |
| 1-stearoyl-GPC (18:0) | -0.05493 | 13-methylmyristate (i15:0) | -0.0429 |
| 2-palmitoyl-GPC (16:0) | -0.05455 | carotene diol (3) | -0.04258 |
| N6,N6,N6-trimethyllysine | -0.05452 | beta-cryptoxanthin | -0.04237 |
| aspartate | -0.05441 | N6-formyllysine | -0.04231 |
| equol sulfate | -0.05434 | N-oleoylglycine | -0.04215 |
| 2-isopropylmalate | -0.05427 | 2-hydroxypalmitate | -0.04213 |
| 1-stearoyl-2-oleoyl-GPC (18:0/18:1) | -0.05425 | N6-carboxyethyllysine | -0.04148 |
| alpha-hydroxyisovalerate | -0.05323 | 5-dodecenoate (12:1n7) | -0.04036 |
| gamma-glutamylglutamine | -0.05311 | 5alpha-pregnan-3beta-ol,20-one sulfate | -0.04019 |
| chenodeoxycholate | -0.0531 | hexadecanedioate (C16) | -0.03996 |
| pheophytin A | -0.05308 | pregnen-diol disulfate | -0.03964 |
| hydroxyproline | -0.05299 | N-butyroyl-sphingosine (d18:1/4:0) | -0.03949 |
| N6-formyllysine | -0.05284 | heptadecasphingosine (d17:1) | -0.03914 |
| 3'-dephospho-acetyl-CoA | -0.05279 | isoursodeoxycholate | -0.03889 |
| UMP | -0.05229 | stigmasterol | -0.03866 |
| 1-oleoyl-GPC (18:1) | -0.0522 | carotene diol (1) | -0.03864 |
| 2-palmitoyl-galactosylglycerol (16:0) | -0.05199 | quinolinate | -0.0385 |
| hexanoylglycine (C6) | -0.05188 | 5alpha-pregnan-3beta,20beta-diol monosulfate (2) | -0.03819 |
| 2-hydroxyheptanoate | -0.05123 | 2-hydroxydecanoate | -0.03817 |
| dimethylarginine (ADMA + SDMA) | -0.05123 | cholesterol | -0.03757 |
| glycine | -0.05108 | N-palmitoyl-sphinganine (d18:0/16:0) | -0.03683 |
| N-acetylfelinine | -0.0509 | 15-methylpalmitate (i17:0) | -0.03677 |
| N-stearoyltaurine | -0.05073 | myristoleate (14:1n5) | -0.03637 |
| salicylate | -0.05042 | 5alpha-pregnan-3beta,20beta-diol monosulfate (1) | -0.03506 |
| 1-linoleoyl-GPE (18:2) | -0.04966 | ceramide (d18:1/17:0, d17:1/18:0) | -0.0338 |
| N-formylmethionine | -0.04964 | N-palmitoyl-heptadecasphingosine (d17:1/16:0) | -0.03358 |
| anserine | -0.04956 | L-urobilin | -0.03338 |
| 1-(1-enyl-stearoyl)-2-arachidonoyl-GPE (P-18:0/20:4) | -0.04951 | hexadecasphingosine (d16:1) | -0.03257 |
| dCMP | -0.04911 | sphingosine | -0.03251 |
| TMP | -0.04901 | N-trimethyl 5-aminovalerate | -0.03243 |
| 1-palmitoyl-2-arachidonoyl-GPC (16:0/20:4n6) | -0.04886 | dodecanedioate (C12) | -0.03172 |
| creatinine | -0.04823 | ursodeoxycholate | -0.03157 |
| caffeic acid sulfate | -0.04805 | N-palmitoylglycine | -0.03115 |
| myo-inositol | -0.04801 | 5alpha-pregnan-3beta,20beta-diol disulfate | -0.03094 |
| 1-linoleoyl-GPC (18:2) | -0.0479 | sphinganine | -0.03024 |
| 1,2-dipalmitoyl-GPE (16:0/16:0) | -0.0479 | campesterol | -0.03019 |
| N-acetyl-cadaverine | -0.04779 | phytosphingosine | -0.03007 |
| gamma-glutamylmethionine | -0.04774 | eicosenoylcarnitine (C20:1) | -0.03 |
| 5-methylcytosine | -0.04756 | 1-methylhistidine | -0.02995 |
| orotidine | -0.04751 | eicosanoylsphingosine (d20:1) | -0.02966 |
| 1-palmitoyl-2-stearoyl-GPC (16:0/18:0) | -0.04751 | 2-aminoadipate | -0.02908 |
| 2,3-dihydroxy-2-methylbutyrate | -0.04749 | 3-methylhistidine | -0.029 |
| glutamate, gamma-methyl ester | -0.04736 | pyridoxamine | -0.02866 |
| lactosyl-N-palmitoyl-sphingosine (d18:1/16:0) | -0.04727 | N-acetylasparagine | -0.02866 |
| 1-linoleoyl-2-arachidonoyl-GPC (18:2/20:4n6) | -0.04685 | serotonin | -0.02851 |
| 2-hydroxyhippurate (salicylurate) | -0.04685 | beta-alanine | -0.02828 |
| 2'-deoxyadenosine 5'-monophosphate | -0.04665 | N-(2-hydroxypalmitoyl)-sphingosine (d18:1/16:0(2OH)) | -0.02799 |
| CMP | -0.04664 | 7-ketolithocholate | -0.02777 |
| N-palmitoyltaurine | -0.04653 | lithocholate | -0.0276 |
| tauroursodeoxycholate | -0.04636 | 3-ureidoisobutyrate | -0.02745 |
| 2-oxoarginine | -0.04622 | ceramide (d18:1/14:0, d16:1/16:0) | -0.02735 |
| taurochenodeoxycholate | -0.04613 | tetradecanedioate (C14) | -0.02713 |
| imidazole lactate | -0.04603 | N-stearoyl-sphingosine (d18:1/18:0) | -0.0266 |
| norvaline | -0.04581 | hexadecenedioate (C16:1-DC) | -0.02635 |
| 1-stearoyl-GPE (18:0) | -0.04568 | dodecenedioate (C12:1-DC) | -0.02629 |
| N-carbamoylalanine | -0.04528 | eicosapentaenoate (EPA; 20:5n3) | -0.02622 |
| hyocholate | -0.04495 | 1-methylguanidine | -0.02616 |
| N-acetylglutamate | -0.04468 | indole | -0.02599 |
| stearoyl sphingomyelin (d18:1/18:0) | -0.04454 | 5alpha-pregnan-3alpha,20beta-diol disulfate | -0.02533 |
| glucosamine 6-sulfate | -0.0444 | beta-sitosterol | -0.02519 |
| betaine | -0.04431 | hydantoin-5-propionic acid | -0.02502 |
| palmitoylcarnitine (C16) | -0.04414 | 3beta,7beta-dihydroxy-5-cholestenoate | -0.02492 |
| valerate (5:0) | -0.04413 | lactosyl-N-palmitoyl-sphingosine (d18:1/16:0) | -0.02458 |
| alpha-CEHC sulfate | -0.0437 | diacetylspermidine | -0.02443 |
| 1-(1-enyl-stearoyl)-2-linoleoyl-GPE (P-18:0/18:2) | -0.04352 | N-stearoyl-sphinganine (d18:0/18:0) | -0.02433 |
| O-sulfo-L-tyrosine | -0.04347 | pregnenolone sulfate | -0.02391 |
| 4-hydroxycinnamate sulfate | -0.04343 | indole-3-carboxylic acid | -0.02368 |
| nicotianamine | -0.04332 | arachidoylcarnitine (C20) | -0.02348 |
| quinate | -0.04314 | pyridoxate | -0.02313 |
| 3-hydroxyhexanoate | -0.043 | trans-nonadecenoate (tr 19:1) | -0.02296 |
| N2-acetyllysine | -0.04268 | 7alpha-hydroxycholestenone | -0.02259 |
| maltotetraose | -0.04248 | retinol (Vitamin A) | -0.02248 |
| histidine betaine (hercynine) | -0.04248 | gamma-glutamylmethionine | -0.02222 |
| N-acetylhistamine | -0.04237 | N-oleoyl-sphingosine (d18:1/18:1) | -0.02221 |
| gamma-glutamyl-epsilon-lysine | -0.04235 | dehydrolithocholate | -0.02199 |
| N-acetylcitrulline | -0.04191 | pterin | -0.02198 |
| histamine | -0.0418 | 3-hydroxyoctanoate | -0.02196 |
| 1-palmitoyl-GPG (16:0) | -0.04169 | deoxycholate | -0.02196 |
| ferulic acid 4-sulfate | -0.04161 | N-acetylsphingosine | -0.02184 |
| genistein | -0.04147 | isovalerylphenylalanine | -0.0213 |
| N-acetylglucosaminylasparagine | -0.04138 | N-acetylputrescine | -0.02106 |
| N-acetylarginine | -0.04134 | anserine | -0.02105 |
| erythritol | -0.04112 | pheophytin A | -0.02081 |
| 2-aminoheptanoate | -0.0411 | glycitein | -0.02055 |
| methionine sulfone | -0.04096 | alpha-tocopherol | -0.02053 |
| taurine | -0.04073 | 2-isopropylmalate | -0.02041 |
| butyrylglycine (C4) | -0.04034 | 2-hydroxyheptanoate | -0.02023 |
| 4-methylcatechol sulfate | -0.04029 | eriodictyol | -0.02012 |
| phenyllactate (PLA) | -0.04025 | 3-ureidopropionate | -0.01975 |
| tyramine O-sulfate | -0.0401 | equol sulfate | -0.01971 |
| 1-stearoyl-GPS (18:0) | -0.03995 | N-hexanoyl-sphingosine (d18:1/6:0) | -0.0197 |
| phenol sulfate | -0.03994 | gamma-CEHC | -0.01965 |
| N-acetylaspartate (NAA) | -0.03993 | 7,12-diketolithocholate | -0.01892 |
| isovalerate (C5) | -0.03974 | tyrosol | -0.01851 |
| betonicine | -0.03943 | gamma-glutamylalanine | -0.01847 |
| N-acetylglucosamine 6-sulfate | -0.03927 | naringenin | -0.0183 |
| threonate | -0.03881 | gamma-glutamylisoleucine | -0.01815 |
| N-acetylserine | -0.03877 | alpha-CEHC | -0.01812 |
| 1-methyladenine | -0.03873 | octadecenedioate (C18:1-DC) | -0.01805 |
| 1-methyl-4-imidazoleacetate | -0.03872 | isoleucine | -0.01798 |
| taurochenodeoxycholate sulfate | -0.03868 | ceramide (d18:1/20:0, d16:1/22:0, d20:1/18:0) | -0.01781 |
| mannitol/sorbitol | -0.03828 | octadecanedioylcarnitine (C18-DC) | -0.01768 |
| phenylacetylglycine | -0.03824 | 3-hydroxydecanoate | -0.01752 |
| naringenin | -0.03792 | hyocholate | -0.01707 |
| gulonate | -0.03781 | 2-hydroxystearate | -0.01692 |
| alpha-hydroxyisocaproate | -0.03758 | 2-oxoarginine | -0.01654 |
| N-oleoylserine | -0.0374 | nervonate (24:1n9) | -0.01546 |
| catechol sulfate | -0.03707 | argininosuccinate | -0.01507 |
| gamma-glutamylalanine | -0.03701 | N6-carboxymethyllysine | -0.01501 |
| glucose | -0.03677 | 2-methylcitrate/homocitrate | -0.01497 |
| ergothioneine | -0.03659 | nicotianamine | -0.01489 |
| cadaverine | -0.0365 | 3-hydroxylaurate | -0.01475 |
| N6-carboxyethyllysine | -0.03641 | hexadecasphinganine (d16:0) | -0.01442 |
| palmitoyl dihydrosphingomyelin (d18:0/16:0) | -0.03637 | stearoyl ethanolamide | -0.01422 |
| carnosine | -0.03626 | docosapentaenoate (n6 DPA; 22:5n6) | -0.01398 |
| prolylglycine | -0.03608 | o-Tyrosine | -0.01395 |
| pyrraline | -0.03576 | I-urobilinogen | -0.01391 |
| taurocholate | -0.03576 | 3-methyl-2-oxovalerate | -0.01363 |
| 5,6-dihydrothymine | -0.0357 | 3-methyl-2-oxobutyrate | -0.01358 |
| dihydroorotate | -0.03552 | margaroyl ethanolamide | -0.01338 |
| felinine | -0.03519 | tricarballylate | -0.01326 |
| indoleacetylglycine | -0.03439 | docosapentaenoylcarnitine (C22:5n3) | -0.01314 |
| pipecolate | -0.03426 | malonate | -0.01305 |
| N-hexanoyl-sphingosine (d18:1/6:0) | -0.03417 | N-palmitoyl-sphingosine (d18:1/16:0) | -0.01303 |
| glycerol 3-phosphate | -0.03417 | beta-muricholate | -0.01299 |
| taurocholenate sulfate | -0.03397 | 7-methylguanine | -0.01271 |
| 2-hydroxy-3-methylvalerate | -0.03347 | pregnanolone/allopregnanolone sulfate | -0.01261 |
| 4-hydroxyhippurate | -0.03328 | N-behenoyl-sphingadienine (d18:2/22:0) | -0.0126 |
| 1-(1-enyl-palmitoyl)-2-oleoyl-GPC (P-16:0/18:1) | -0.03324 | urate | -0.01226 |
| bilirubin (E,Z or Z,E) | -0.03312 | taurodeoxycholate | -0.01201 |
| 3-(4-hydroxyphenyl)lactate (HPLA) | -0.03304 | 10-hydroxystearate | -0.01193 |
| glycerophosphoserine | -0.03249 | octadecenedioylcarnitine (C18:1-DC) | -0.01187 |
| cinnamoylglycine | -0.03245 | N-acetylglutamate | -0.01164 |
| argininate | -0.03238 | 4-acetamidobutanoate | -0.0116 |
| 4-imidazoleacetate | -0.03195 | N1,N12-diacetylspermine | -0.01148 |
| putrescine | -0.03164 | 4-hydroxyphenylpyruvate | -0.01115 |
| 2-stearoyl-GPE (18:0) | -0.03162 | 1-methylxanthine | -0.01113 |
| sphingomyelin (d18:1/17:0, d17:1/18:0, d19:1/16:0) | -0.03161 | 2,3-dihydroxyisovalerate | -0.01098 |
| valerylglycine (C5) | -0.03157 | cytosine | -0.01098 |
| taurolithocholate 3-sulfate | -0.03153 | N-stearoyltaurine | -0.01065 |
| FAD | -0.03152 | 2-aminoheptanoate | -0.01056 |
| nicotinic acid mononucleotide (NaMN) | -0.03146 | genistein | -0.01055 |
| alanine | -0.03141 | stearoylcarnitine (C18) | -0.01045 |
| ribitol | -0.03091 | 6-oxopiperidine-2-carboxylate | -0.01039 |
| vanillate | -0.0309 | trimethylamine N-oxide | -0.01038 |
| 1-methylhistamine | -0.03073 | N-acetyl-1-methylhistidine | -0.01012 |
| 1,2-dipalmitoyl-GPC (16:0/16:0) | -0.03072 | pyruvate | -0.00976 |
| N6-acetyllysine | -0.03067 | alpha-hydroxycaproate | -0.00971 |
| N-methylhydroxyproline | -0.03049 | kynurenate | -0.00951 |
| 5,6-dihydrouracil | -0.03038 | caproate (6:0) | -0.0089 |
| phenylpropionylglycine | -0.03036 | 5-methylcytosine | -0.00874 |
| glutarate (C5-DC) | -0.03016 | 2-(4-hydroxyphenyl)propionate | -0.00854 |
| p-cresol sulfate | -0.03008 | feruloylputrescine | -0.00838 |
| N-acetylmethionine | -0.02969 | malate | -0.00832 |
| glycerophosphoinositol | -0.02943 | norvaline | -0.00825 |
| sinapate | -0.02924 | pipecolate | -0.00809 |
| 1-oleoyl-GPE (18:1) | -0.02919 | indolin-2-one | -0.00784 |
| 1-(1-enyl-stearoyl)-GPE (P-18:0) | -0.02905 | erucate (22:1n9) | -0.00765 |
| 2-hydroxyglutarate | -0.02898 | palmitoylcarnitine (C16) | -0.0075 |
| cysteine sulfinic acid | -0.02898 | lactate | -0.00743 |
| N-acetylputrescine | -0.02894 | valine | -0.00735 |
| 1-palmitoyl-2-arachidonoyl-GPE (16:0/20:4) | -0.02863 | 4-methyl-2-oxopentanoate | -0.00729 |
| oleanolate | -0.0285 | cysteine s-sulfate | -0.00727 |
| 3-hydroxybutyrate (BHBA) | -0.02826 | 6-hydroxynicotinate | -0.00719 |
| thiamin monophosphate | -0.02808 | tyrosine | -0.00671 |
| sedoheptulose | -0.02766 | maltose | -0.00665 |
| 1-(1-enyl-palmitoyl)-2-linoleoyl-GPE (P-16:0/18:2) | -0.02764 | daidzein | -0.00662 |
| N-oleoyltaurine | -0.0276 | 3-sulfo-L-alanine | -0.00659 |
| 1-palmitoyl-2-docosahexaenoyl-GPC (16:0/22:6) | -0.02723 | D-urobilin | -0.00653 |
| N-acetyltaurine | -0.02709 | docosahexaenoate (DHA; 22:6n3) | -0.00649 |
| N-acetylkynurenine (2) | -0.02707 | 3-hydroxyhexanoate | -0.00648 |
| 1-palmitoyl-2-oleoyl-GPE (16:0/18:1) | -0.02703 | 3-dehydroshikimate | -0.00613 |
| ribulose/xylulose | -0.02702 | maltotriose | -0.00605 |
| lactate | -0.02669 | 3-hydroxybutyrylcarnitine (2) | -0.00601 |
| N-stearoylserine | -0.02651 | 2-hydroxybehenate | -0.00595 |
| 6-oxopiperidine-2-carboxylate | -0.02643 | phenylacetate | -0.00589 |
| N-acetylalanine | -0.02643 | carnitine | -0.00581 |
| hippurate | -0.02606 | 3-hydroxyoleate | -0.00569 |
| 5-oxoproline | -0.02599 | dihomolinoleate (20:2n6) | -0.00564 |
| hydantoin-5-propionic acid | -0.02599 | indolepropionate | -0.00563 |
| arabonate/xylonate | -0.02584 | 2-hydroxy-3-methylvalerate | -0.00539 |
| 2-(4-hydroxyphenyl)propionate | -0.02566 | N-acetylarginine | -0.00526 |
| 4-vinylguaiacol sulfate | -0.02549 | 17-methylstearate (i19:0) | -0.00517 |
| palmitoylcholine | -0.02548 | methionine sulfoxide | -0.0051 |
| indolelactate | -0.0253 | alpha-ketoglutarate | -0.0051 |
| threonine | -0.02527 | 2-hydroxyhippurate (salicylurate) | -0.00496 |
| 2'-O-methyluridine | -0.0252 | 3'-dephospho-acetyl-CoA | -0.00485 |
| N-acetylmethionine sulfoxide | -0.02509 | arachidonoyl ethanolamide | -0.00485 |
| 1-ribosyl-imidazoleacetate | -0.02499 | docosadienoate (22:2n6) | -0.00433 |
| dimethylglycine | -0.02487 | docosapentaenoate (DPA; 22:5n3) | -0.00426 |
| arabitol/xylitol | -0.02426 | indolelactate | -0.00424 |
| methyl indole-3-acetate | -0.02425 | alpha-hydroxyisovalerate | -0.00415 |
| suberate (C8-DC) | -0.0242 | 3-(4-hydroxyphenyl)lactate (HPLA) | -0.00397 |
| glutamate | -0.02403 | oleanolate | -0.0039 |
| cholate | -0.02397 | nicotinic acid mononucleotide (NaMN) | -0.00387 |
| homoserine (homoserine lactone) | -0.02388 | 2-hydroxynervonate | -0.0037 |
| quinolinate | -0.02358 | arginine | -0.00329 |
| indoleacetate | -0.02346 | N-formylphenylalanine | -0.00324 |
| proline | -0.02327 | lysine | -0.00324 |
| N-acetylthreonine | -0.02314 | N2-acetyllysine | -0.00313 |
| 1-palmitoyl-GPE (16:0) | -0.02308 | p-cresol | -0.00292 |
| N-acetylneuraminate | -0.02299 | phenethylamine | -0.00286 |
| 2-hydroxybutyrate/2-hydroxyisobutyrate | -0.02293 | N-palmitoyltaurine | -0.00274 |
| 3-(3-hydroxyphenyl)propionate sulfate | -0.02281 | orotate | -0.00259 |
| N6-carboxymethyllysine | -0.0228 | 3-hydroxybutyrate (BHBA) | -0.0021 |
| pseudouridine | -0.02248 | imidazole lactate | -0.00204 |
| allantoin | -0.02208 | 2-hydroxyglutarate | -0.00181 |
| 3-dehydroshikimate | -0.0219 | 3-hydroxyadipate | -0.00176 |
| N-linoleoyltaurine | -0.0219 | 2-hydroxyadipate | -0.00174 |
| oleoylcholine | -0.02179 | 1-methyl-4-imidazoleacetate | -0.00168 |
| glycerophosphoethanolamine | -0.02175 | behenate (22:0) | -0.00155 |
| glycocholate | -0.02152 | picolinate | -0.00143 |
| glycerophosphorylcholine (GPC) | -0.0214 | phenylpropionylglycine | -0.00126 |
| glycerophosphoglycerol | -0.02129 | piperidine | -0.00121 |
| ribonate (ribonolactone) | -0.02108 | heptanoate (7:0) | -0.00113 |
| N-palmitoleoyltaurine | -0.02086 | alpha-tocotrienol | -0.0011 |
| agmatine | -0.02078 | glutarate (C5-DC) | -0.0009 |
| pimelate (C7-DC) | -0.02061 | cystine | -0.00078 |
| margaroyl ethanolamide | -0.02057 | isovalerylcarnitine (C5) | -0.00069 |
| glutamine | -0.02032 | saccharin | -0.00067 |
| N-acetylcysteine | -0.02029 | bilirubin (E,E) | -0.00064 |
| benzoate | -0.02023 | 5-hydroxyhexanoate | -0.00063 |
| N-acetylhistidine | -0.02016 | sphingomyelin (d18:0/18:0, d19:0/17:0) | -0.0006 |
| sarcosine | -0.02007 | erucoylcarnitine (C22:1) | -0.00021 |
| N-trimethyl 5-aminovalerate | -0.01934 | phosphocholine | 0.00007 |
| N(1)-acetylspermine | -0.01928 | carnosine | 0.00019 |
| pheophorbide A | -0.01909 | bilirubin (E,Z or Z,E) | 0.00047 |
| methionine | -0.01905 | PAHSA (16:0/OH-18:0) | 0.00052 |
| N-acetyl-1-methylhistidine | -0.01903 | valeryltryptophan | 0.00066 |
| palmitoyl sphingomyelin (d18:1/16:0) | -0.01896 | cis-urocanate | 0.00081 |
| erythronate | -0.01882 | N-alpha-acetylornithine | 0.00087 |
| diacetylspermidine | -0.01876 | genistein sulfate | 0.00103 |
| syringic acid | -0.01853 | leucine | 0.0011 |
| laurylcarnitine (C12) | -0.01845 | alpha-tocopherol acetate | 0.00112 |
| 5-hydroxylysine | -0.0184 | bilirubin | 0.00116 |
| thioproline | -0.01821 | dihydroorotate | 0.00117 |
| 2,3-dihydroxyisovalerate | -0.01764 | sphingadienine | 0.00118 |
| deoxymugineic acid | -0.01761 | trans-urocanate | 0.00133 |
| stearoylcholine | -0.01756 | NAD+ | 0.00142 |
| cysteinylglycine | -0.01745 | daidzein sulfate (2) | 0.00145 |
| malate | -0.01714 | citrate | 0.00202 |
| creatine | -0.01713 | mannitol/sorbitol | 0.00212 |
| 3-hydroxydecanoate | -0.01702 | N-acetylcysteine | 0.00213 |
| 1-linoleoyl-GPG (18:2) | -0.01666 | enterolactone | 0.00217 |
| 1-palmitoyl-digalactosylglycerol (16:0) | -0.0165 | 4-hydroxyhippurate | 0.00234 |
| 1-stearoyl-GPI (18:0) | -0.01632 | N(1)-acetylspermine | 0.00242 |
| stearoyl ethanolamide | -0.01621 | 5-(galactosylhydroxy)-L-lysine | 0.00246 |
| valerylphenylalanine | -0.01614 | tauroursodeoxycholate | 0.00254 |
| cysteine | -0.01607 | acetylcarnitine (C2) | 0.0026 |
| phosphocholine | -0.01564 | FAD | 0.00316 |
| cytidine 2',3'-cyclic monophosphate | -0.01552 | sulfate | 0.00326 |
| pyruvate | -0.01547 | adenine | 0.00339 |
| dodecenedioate (C12:1-DC) | -0.01538 | pantothenate (Vitamin B5) | 0.00344 |
| galactitol (dulcitol) | -0.01537 | glutamate, gamma-methyl ester | 0.00355 |
| 1-palmitoyl-2-oleoyl-GPC (16:0/18:1) | -0.01511 | N-formylanthranilic acid | 0.0036 |
| 3-hydroxyoctanoate | -0.01508 | phenylpyruvate | 0.00371 |
| N-propionylmethionine | -0.01455 | aspartate | 0.00384 |
| 1-methylguanidine | -0.01437 | lanosterol | 0.00387 |
| argininosuccinate | -0.01412 | hexadecadienoate (16:2n6) | 0.00397 |
| glycosyl-N-palmitoyl-sphingosine (d18:1/16:0) | -0.01403 | alpha-hydroxyisocaproate | 0.00418 |
| 3-sulfo-L-alanine | -0.01399 | 4-hydroxybutyrate (GHB) | 0.0042 |
| 3-(4-hydroxyphenyl)propionate | -0.01392 | indoleacetylglycine | 0.00433 |
| oleoylcarnitine (C18) | -0.0139 | betonicine | 0.00452 |
| 2-aminophenol sulfate | -0.01361 | 5-methylthioadenosine (MTA) | 0.00475 |
| eicosapentaenoate (EPA; 20:5n3) | -0.01361 | 2-palmitoyl-galactosylglycerol (16:0) | 0.00484 |
| ursocholate | -0.01338 | dimethylarginine (ADMA + SDMA) | 0.00488 |
| 4-hydroxybenzoate | -0.01332 | 1-(1-enyl-palmitoyl)-2-linoleoyl-GPE (P-16:0/18:2) | 0.00493 |
| heptanoate (7:0) | -0.01322 | adrenate (22:4n6) | 0.00509 |
| N-delta-acetylornithine | -0.01287 | allo-threonine | 0.00514 |
| N-glycolylneuraminate | -0.01276 | 2-hydroxyphenylacetate | 0.00535 |
| 4-hydroxybutyrate (GHB) | -0.01276 | 5-hydroxylysine | 0.00541 |
| 1-oleoyl-GPG (18:1) | -0.0126 | 2-hydroxyarachidate | 0.00547 |
| 5-hydroxydecanoate | -0.01255 | coprostanol | 0.00569 |
| methionine sulfoxide | -0.0125 | argininate | 0.00598 |
| spermidine | -0.01221 | N-acetylphenylalanine | 0.00602 |
| phenylpyruvate | -0.01202 | 5-hydroxydecanoate | 0.00615 |
| diacetylchitobiose | -0.01182 | methyl indole-3-acetate | 0.00618 |
| 5-hydroxymethylcytosine | -0.01164 | 3-ketosphinganine | 0.0064 |
| succinate | -0.0116 | quinate | 0.00645 |
| 8-hydroxyguanine | -0.01158 | N-acetylisoleucine | 0.00659 |
| glycosyl ceramide (d18:1/20:0, d16:1/22:0) | -0.01113 | N6-acetyllysine | 0.00701 |
| N-acetyl-beta-alanine | -0.01113 | 4-imidazoleacetate | 0.0071 |
| ethylmalonate | -0.01099 | steviol | 0.0072 |
| N-butyroyl-sphingosine (d18:1/4:0) | -0.0105 | hexanoylglycine (C6) | 0.00726 |
| glucuronate | -0.00988 | N-methylhydantoin | 0.00726 |
| methylsuccinate | -0.00959 | 1-palmitoyl-galactosylglycerol (16:0) | 0.00727 |
| uridine-2',3'-cyclic monophosphate | -0.00955 | 3-dehydrocholate | 0.0073 |
| homogentisate | -0.00953 | 2-hydroxybutyrate/2-hydroxyisobutyrate | 0.00732 |
| allo-threonine | -0.00941 | succinimide | 0.00744 |
| 3-methyl-2-oxobutyrate | -0.00926 | N-acetyltyrosine | 0.00745 |
| oleoyl ethanolamide | -0.00915 | 1-methyl-beta-carboline-3-carboxylic acid | 0.00758 |
| mevalonolactone | -0.0087 | glycocholate | 0.00761 |
| citrate | -0.00848 | 9,10-DiHOME | 0.00767 |
| glycerate | -0.00837 | indoleacetate | 0.00775 |
| beta-cryptoxanthin | -0.00832 | 5-(2-Hydroxyethyl)-4-methylthiazole | 0.00806 |
| sphingomyelin (d18:1/24:1, d18:2/24:0) | -0.00826 | 12,13-DiHOME | 0.00809 |
| N-methylhydantoin | -0.00813 | deoxymugineic acid | 0.00819 |
| hydroxymethylpyrimidine | -0.00809 | serine | 0.00825 |
| 1-stearoyl-GPG (18:0) | -0.008 | gamma-glutamylglutamine | 0.0085 |
| 5-(2-Hydroxyethyl)-4-methylthiazole | -0.00788 | betaine | 0.00864 |
| tyramine | -0.00747 | N-stearoylserine | 0.00907 |
| bilirubin (E,E) | -0.00694 | N-acetylglucosaminylasparagine | 0.00909 |
| picolinate | -0.00691 | 8-hydroxyguanine | 0.00925 |
| 2-hydroxydecanoate | -0.00668 | biliverdin | 0.00962 |
| bilirubin | -0.00648 | 7-ketodeoxycholate | 0.00972 |
| N-acetylisoleucine | -0.00646 | phosphate | 0.00984 |
| dihydroferulic acid | -0.00642 | alpha-ketoglutaramate | 0.00986 |
| beta-hydroxyisovalerate | -0.0064 | taurochenodeoxycholate | 0.01 |
| mevalonate | -0.00626 | N-acetyl-cadaverine | 0.01007 |
| myristoylcarnitine (C14) | -0.00618 | ethylmalonate | 0.01024 |
| 5-hydroxyhexanoate | -0.00603 | phenylacetylglycine | 0.01038 |
| alpha-ketoglutarate | -0.00487 | 2,3-dihydroxy-2-methylbutyrate | 0.01041 |
| cholate sulfate | -0.0048 | phenylalanine | 0.01058 |
| sphingomyelin (d18:1/14:0, d16:1/16:0) | -0.00478 | 1,2-dipalmitoyl-GPE (16:0/16:0) | 0.0106 |
| N-monomethylarginine | -0.00471 | N-acetylaspartate (NAA) | 0.01073 |
| gentisate | -0.00466 | AMP | 0.01093 |
| N-acetylphenylalanine | -0.00423 | xanthurenate | 0.01122 |
| adipate | -0.00416 | 4-hydroxyphenylacetate | 0.01128 |
| 3-hydroxylaurate | -0.0039 | deoxycholic acid sulfate | 0.01136 |
| cysteine s-sulfate | -0.00389 | OAHSA (18:1/OH-18:0) | 0.01151 |
| 1-(1-enyl-oleoyl)-GPE (P-18:1) | -0.00344 | pheophorbide A | 0.01152 |
| N1,N12-diacetylspermine | -0.00299 | chenodeoxycholate | 0.01159 |
| 3-hydroxyisobutyrate | -0.00298 | hydroxymethylpyrimidine | 0.0119 |
| caprylate (8:0) | -0.00285 | laurylcarnitine (C12) | 0.01196 |
| N-acetyltryptophan | -0.00257 | N-methylalanine | 0.01207 |
| 1,5-anhydroglucitol (1,5-AG) | -0.00229 | N-acetylserine | 0.01218 |
| 4-methyl-2-oxopentanoate | -0.00219 | pimelate (C7-DC) | 0.01222 |
| piperidine | -0.00189 | isovalerylglycine | 0.01252 |
| 2'-deoxycytidine | -0.0016 | caffeate | 0.01262 |
| sulfate | -0.00154 | palmitoleoyl ethanolamide | 0.01266 |
| tryptamine | -0.00134 | N-acetylcitrulline | 0.01277 |
| 2-methylcitrate/homocitrate | -0.00119 | valerylphenylalanine | 0.01293 |
| 3-hydroxyphenylacetate | -0.00106 | suberate (C8-DC) | 0.013 |
| docosapentaenoate (n6 DPA; 22:5n6) | -0.00086 | CMP | 0.01318 |
| trans-nonadecenoate (tr 19:1) | -0.00069 | N-oleoylserine | 0.01319 |
| 2-piperidinone | 0.0004 | 1-(1-enyl-stearoyl)-2-arachidonoyl-GPE (P-18:0/20:4) | 0.01319 |
| kynurenine | 0.00058 | methylmalonate (MMA) | 0.0132 |
| N-acetylleucine | 0.00067 | 4-cholesten-3-one | 0.01322 |
| sebacate (C10-DC) | 0.00075 | N-acetylglutamine | 0.01346 |
| equol | 0.00098 | glycylisoleucine | 0.01349 |
| 5-aminovalerate | 0.0011 | palmitoleoylcarnitine (C16:1) | 0.01349 |
| isovalerylglycine | 0.00115 | N-acetyl-beta-alanine | 0.01384 |
| tryptophan | 0.00132 | margarate (17:0) | 0.014 |
| guanine | 0.00137 | 2-palmitoyl-GPC (16:0) | 0.01419 |
| N-acetylglutamine | 0.00138 | glucosamine 6-sulfate | 0.0144 |
| adrenate (22:4n6) | 0.00141 | taurocholate | 0.01479 |
| 3-methyl-2-oxovalerate | 0.00148 | cysteine sulfinic acid | 0.01506 |
| N-acetylglucosamine/N-acetylgalactosamine | 0.00177 | N-acetyl-isoputreanine | 0.01523 |
| carotene diol (2) | 0.00178 | ferulic acid 4-sulfate | 0.01535 |
| stachydrine | 0.00198 | dihomolinolenate (20:3n3 or 3n6) | 0.01555 |
| hydroquinone sulfate | 0.00204 | 3-phenylpropionate (hydrocinnamate) | 0.0156 |
| alpha-tocopherol acetate | 0.00215 | N-delta-acetylornithine | 0.01581 |
| kynurenate | 0.00241 | 1,5-anhydroglucitol (1,5-AG) | 0.01589 |
| 1-palmitoyl-2-palmitoleoyl-GPC (16:0/16:1) | 0.00249 | threonine | 0.01592 |
| mead acid (20:3n9) | 0.00251 | nonadecanoate (19:0) | 0.01601 |
| histidine | 0.00255 | 3-aminoisobutyrate | 0.01608 |
| 17alpha-hydroxypregnenolone 3-sulfate | 0.00294 | maltotetraose | 0.01611 |
| ornithine | 0.00328 | 4-methylcatechol sulfate | 0.01618 |
| feruloylputrescine | 0.00359 | 5-oxoproline | 0.01622 |
| caffeate | 0.00424 | N-palmitoleoyltaurine | 0.01629 |
| linoleoylcarnitine (C18:2) | 0.00433 | 1-stearoyl-2-arachidonoyl-GPE (18:0/20:4) | 0.0163 |
| N-acetyltyrosine | 0.00436 | dCMP | 0.01632 |
| 3-methylhistidine | 0.00452 | UMP | 0.01656 |
| glycosyl-N-stearoyl-sphingosine (d18:1/18:0) | 0.00482 | N-acetylhistamine | 0.01656 |
| erythrose | 0.00487 | caprylate (8:0) | 0.01667 |
| carnitine | 0.0051 | alpha-CEHC sulfate | 0.01678 |
| 2-acetamidobutanoate | 0.00533 | cinnamoylglycine | 0.01685 |
| steviol | 0.00558 | citrulline | 0.01688 |
| xanthine | 0.00584 | phenyllactate (PLA) | 0.01689 |
| p-cresol | 0.00613 | matairesinol | 0.0169 |
| 5-dodecenoylcarnitine (C12:1) | 0.00615 | 12-dehydrocholate | 0.01721 |
| pyroglutamine | 0.00628 | cytidine 2',3'-cyclic monophosphate | 0.01756 |
| N-behenoyl-sphingadienine (d18:2/22:0) | 0.00663 | 4-hydroxybenzoate | 0.0176 |
| saccharin | 0.00705 | glycerophosphorylcholine (GPC) | 0.01775 |
| docosahexaenoate (DHA; 22:6n3) | 0.00726 | ceramide (d18:2/24:1, d18:1/24:2) | 0.01784 |
| DIMBOA | 0.00733 | tryptophan | 0.01792 |
| 4-hydroxyphenylpyruvate | 0.00769 | hippurate | 0.0181 |
| 2'-deoxyguanosine | 0.00796 | glutamate | 0.01827 |
| riboflavin (Vitamin B2) | 0.00802 | N-acetylneuraminate | 0.01839 |
| linoleoyl-docosahexaenoyl-glycerol (18:2/22:6) [2] | 0.00813 | ornithine | 0.01902 |
| nicotinamide | 0.00864 | N-acetylglycine | 0.01944 |
| eicosenoate (20:1n9 or 1n11) | 0.00878 | glycine | 0.01994 |
| myristoleoylcarnitine (C14:1) | 0.0092 | myristoylcarnitine (C14) | 0.01995 |
| 2-hydroxyphenylacetate | 0.00994 | butyrylglycine (C4) | 0.01998 |
| N-palmitoylglycine | 0.01026 | arachidate (20:0) | 0.02004 |
| 4-hydroxyphenylacetate | 0.01031 | N1-Methyl-2-pyridone-5-carboxamide | 0.02005 |
| guanidinoacetate | 0.01058 | taurochenodeoxycholate sulfate | 0.02014 |
| guanosine | 0.01094 | methionine sulfone | 0.02017 |
| 1-methylnicotinamide | 0.01133 | 17alpha-hydroxypregnenolone 3-sulfate | 0.0202 |
| cystine | 0.01143 | 4-hydroxycinnamate sulfate | 0.02023 |
| inosine | 0.01177 | hydroxyproline | 0.02031 |
| lanosterol | 0.01204 | 5,6-dihydrouracil | 0.02033 |
| xanthurenate | 0.01209 | caffeic acid sulfate | 0.02035 |
| malonylcarnitine | 0.01222 | N-acetylleucine | 0.02041 |
| phenylacetate | 0.0125 | gamma-tocotrienol | 0.02053 |
| valeryltryptophan | 0.01328 | deoxycarnitine | 0.02054 |
| taurodeoxycholate | 0.01339 | ursodeoxycholate sulfate (1) | 0.02058 |
| pyridoxal | 0.01356 | ursocholate | 0.02059 |
| 1-methylxanthine | 0.01383 | tryptamine | 0.02078 |
| cytidine | 0.01399 | oleoylcarnitine (C18) | 0.02089 |
| fructose | 0.01414 | enterodiol | 0.02097 |
| biliverdin | 0.01422 | 3-methylglutarate/2-methylglutarate | 0.02103 |
| oleate/vaccenate (18:1) | 0.01432 | N-monomethylarginine | 0.02129 |
| arachidate (20:0) | 0.01434 | thiamin monophosphate | 0.02132 |
| N-acetylmuramate | 0.01438 | valerate (5:0) | 0.0216 |
| thymidine | 0.01456 | isovalerate (C5) | 0.0218 |
| nicotinate ribonucleoside | 0.01461 | 5-methyl-2'-deoxycytidine | 0.02194 |
| 3-methylglutarate/2-methylglutarate | 0.01461 | 2'-deoxyadenosine 5'-monophosphate | 0.02214 |
| N-acetylasparagine | 0.01464 | gulonate | 0.02216 |
| 1-(1-enyl-palmitoyl)-GPE (P-16:0) | 0.01473 | ergothioneine | 0.02223 |
| pyridoxine (Vitamin B6) | 0.01479 | N-methylhydroxyproline | 0.02225 |
| tryptophylglycine | 0.01482 | N-acetyltryptophan | 0.02241 |
| palmitoleoylcarnitine (C16:1) | 0.01497 | 1-palmitoyl-GPC (16:0) | 0.02264 |
| 12-dehydrocholate | 0.01502 | undecanedioate (C11-DC) | 0.02272 |
| trigonelline (N'-methylnicotinate) | 0.01504 | 10-heptadecenoate (17:1n7) | 0.02306 |
| galacturonate | 0.01513 | N-acetylkynurenine (2) | 0.02311 |
| 3-dehydrocholate | 0.01518 | sebacate (C10-DC) | 0.02351 |
| 7-ketodeoxycholate | 0.0152 | N-palmitoylserine | 0.02352 |
| behenate (22:0) | 0.01554 | 1-(1-enyl-stearoyl)-2-linoleoyl-GPE (P-18:0/18:2) | 0.02386 |
| 8-hydroxyoctanoate | 0.0156 | asparagine | 0.02395 |
| adenine | 0.01575 | putrescine | 0.02396 |
| hypoxanthine | 0.01583 | pseudouridine | 0.02404 |
| methylphosphate | 0.01599 | 3-hydroxyphenylacetate | 0.02413 |
| ursodeoxycholate | 0.01609 | palmitate (16:0) | 0.02421 |
| dodecanedioate (C12) | 0.01623 | orotidine | 0.02433 |
| adenosine | 0.01635 | 1-palmitoyl-2-docosahexaenoyl-GPC (16:0/22:6) | 0.02435 |
| beta-guanidinopropanoate | 0.01658 | histamine | 0.02444 |
| isovalerylcarnitine (C5) | 0.01685 | tyramine O-sulfate | 0.02446 |
| 1-oleoyl-2-linoleoyl-GPE (18:1/18:2) | 0.01693 | stearate (18:0) | 0.02461 |
| OAHSA (18:1/OH-18:0) | 0.01703 | eicosenoate (20:1n9 or 1n11) | 0.02474 |
| 2'-deoxyadenosine | 0.01724 | 2-piperidinone | 0.0248 |
| serine | 0.01727 | proline | 0.02484 |
| N-acetylvaline | 0.01735 | N-acetylfelinine | 0.02516 |
| stigmasterol | 0.01786 | tyramine | 0.02518 |
| dipicolinate | 0.01793 | uridine-2',3'-cyclic monophosphate | 0.02543 |
| nicotinamide riboside | 0.01807 | pyroglutamine | 0.02545 |
| fumarate | 0.01833 | erythronate | 0.02607 |
| matairesinol | 0.01836 | histidine | 0.02608 |
| malonate | 0.01854 | linoleoylcarnitine (C18:2) | 0.0266 |
| carotene diol (1) | 0.01871 | cholate sulfate | 0.02671 |
| arabinose | 0.01974 | allantoin | 0.02679 |
| erucate (22:1n9) | 0.0198 | sphingomyelin (d18:1/20:0, d16:1/22:0) | 0.02689 |
| 1,2-dilinoleoyl-digalactosylglycerol (18:2/18:2) | 0.01996 | 4-vinylguaiacol sulfate | 0.02707 |
| N-methylproline | 0.01999 | mevalonolactone | 0.02717 |
| imidazole propionate | 0.02016 | 1-(1-enyl-palmitoyl)-2-oleoyl-GPC (P-16:0/18:1) | 0.02744 |
| 5alpha-pregnan-3beta,20beta-diol monosulfate (1) | 0.02037 | catechol sulfate | 0.02763 |
| anthranilate | 0.02039 | homogentisate | 0.02775 |
| 4-hydroxycinnamate | 0.02049 | N-oleoyltaurine | 0.02782 |
| 2-hydroxyadipate | 0.02067 | ribitol | 0.02787 |
| N-formylphenylalanine | 0.02078 | diacetylchitobiose | 0.02794 |
| nonadecanoate (19:0) | 0.02091 | 3-(4-hydroxyphenyl)propionate | 0.02798 |
| 3-hydroxysuberate | 0.02106 | dimethylglycine | 0.02798 |
| sphingadienine | 0.02131 | beta-guanidinopropanoate | 0.02799 |
| propionylglycine (C3) | 0.02132 | palmitoleate (16:1n7) | 0.028 |
| fucose | 0.02137 | threonate | 0.02815 |
| ferulate | 0.02147 | erythritol | 0.02837 |
| palmitate (16:0) | 0.02151 | glutamine | 0.02847 |
| 1-palmitoyl-2-linoleoyl-GPE (16:0/18:2) | 0.02157 | 2-oxindole-3-acetate | 0.02864 |
| 10-nonadecenoate (19:1n9) | 0.02192 | 3-hydroxysebacate | 0.0287 |
| stearate (18:0) | 0.02225 | TMP | 0.02885 |
| 2'-deoxyuridine | 0.02237 | imidazole propionate | 0.02974 |
| N1-Methyl-2-pyridone-5-carboxamide | 0.02276 | 3-hydroxyisobutyrate | 0.02979 |
| 1-palmitoyl-2-linoleoyl-GPC (16:0/18:2) | 0.02314 | methylphosphate | 0.0299 |
| tricarballylate | 0.02347 | gamma-glutamyl-epsilon-lysine | 0.0302 |
| 7,12-diketolithocholate | 0.02363 | cadaverine | 0.03039 |
| 3-phenylpropionate (hydrocinnamate) | 0.02375 | cholate | 0.03066 |
| lysylleucine | 0.02391 | thiamin (Vitamin B1) | 0.03091 |
| formiminoglutamate | 0.02422 | alanine | 0.03114 |
| dehydrolithocholate | 0.02455 | (N(1) + N(8))-acetylspermidine | 0.03129 |
| 1-palmitoyl-2-linoleoyl-digalactosylglycerol (16:0/18:2) | 0.02464 | N-acetylmethionine sulfoxide | 0.03139 |
| 5alpha-pregnan-3beta-ol,20-one sulfate | 0.02465 | 1-stearoyl-2-arachidonoyl-GPC (18:0/20:4) | 0.03158 |
| valylleucine | 0.02472 | 1-palmitoyl-2-arachidonoyl-GPE (16:0/20:4) | 0.03175 |
| alanylleucine | 0.02479 | N-acetyltaurine | 0.03204 |
| 5-methyl-2'-deoxycytidine | 0.02486 | agmatine | 0.03211 |
| azelate (nonanedioate; C9) | 0.02515 | 2'-deoxyadenosine | 0.03217 |
| N-acetylproline | 0.02524 | 5-dodecenoylcarnitine (C12:1) | 0.03231 |
| leucylalanine | 0.02532 | LAHSA (18:2/OH-18:0) | 0.03253 |
| octadecenedioylcarnitine (C18:1-DC) | 0.02577 | methionine | 0.03255 |
| tyrosol | 0.02585 | 2-acetamidobutanoate | 0.03258 |
| 5,6-dihydrouridine | 0.02593 | phenol sulfate | 0.03265 |
| 3-methylglutaconate | 0.02598 | ribonate (ribonolactone) | 0.03268 |
| secoisolariciresinol diglucoside | 0.02598 | valerylglycine (C5) | 0.03284 |
| nervonate (24:1n9) | 0.0265 | delta-tocopherol | 0.03352 |
| uridine | 0.02666 | prolylglycine | 0.03375 |
| N-alpha-acetylornithine | 0.02693 | 5-hydroxymethylcytosine | 0.03412 |
| 4-guanidinobutanoate | 0.02712 | 1-linoleoyl-2-arachidonoyl-GPC (18:2/20:4n6) | 0.03432 |
| 2'-deoxyinosine | 0.0273 | homoserine (homoserine lactone) | 0.0344 |
| homocitrulline | 0.02734 | p-cresol sulfate | 0.0348 |
| ursodeoxycholate sulfate (1) | 0.02751 | histidine betaine (hercynine) | 0.03494 |
| 3-hydroxyoleate | 0.02752 | 1-stearoyl-2-oleoyl-GPC (18:0/18:1) | 0.03494 |
| PAHSA (16:0/OH-18:0) | 0.0277 | 2'-O-methyluridine | 0.03505 |
| valine | 0.02778 | myo-inositol | 0.03509 |
| 7-ketolithocholate | 0.02788 | N-acetylvaline | 0.03524 |
| arachidonate (20:4n6) | 0.02792 | 8-hydroxyoctanoate | 0.03576 |
| sphingomyelin (d18:0/18:0, d19:0/17:0) | 0.02792 | glycerol 3-phosphate | 0.03587 |
| leucylglutamine | 0.02824 | salicylate | 0.03597 |
| stearoylcarnitine (C18) | 0.0283 | sedoheptulose | 0.0363 |
| mannose | 0.02836 | N-methylproline | 0.03644 |
| 17-methylstearate (i19:0) | 0.02842 | N-acetylproline | 0.03655 |
| 3-aminoisobutyrate | 0.02848 | tryptophylglycine | 0.03657 |
| pyridoxate | 0.0285 | N-propionylmethionine | 0.03666 |
| 2-oxindole-3-acetate | 0.02851 | 13-HODE + 9-HODE | 0.03674 |
| N-oleoylglycine | 0.02869 | N-acetylhistidine | 0.03677 |
| deoxycarnitine | 0.02882 | riboflavin (Vitamin B2) | 0.03684 |
| 3-ureidopropionate | 0.02928 | N6,N6,N6-trimethyllysine | 0.037 |
| acetylcarnitine (C2) | 0.02957 | uracil | 0.0374 |
| (N(1) + N(8))-acetylspermidine | 0.02973 | spermidine | 0.03766 |
| lysine | 0.02984 | glycylvaline | 0.03777 |
| gamma-CEHC | 0.02994 | beta-hydroxyisovalerate | 0.0381 |
| propionylglutamine | 0.03033 | stearoyl sphingomyelin (d18:1/18:0) | 0.0383 |
| N-acetyl-3-methylhistidine | 0.03062 | 1-methyladenine | 0.03847 |
| 4-cholesten-3-one | 0.03092 | linoleoyl-linoleoyl-glycerol (18:2/18:2) [2] | 0.03853 |
| 4-acetamidobutanoate | 0.03107 | diaminopimelate | 0.03863 |
| pyridoxamine | 0.03126 | myristoleoylcarnitine (C14:1) | 0.03873 |
| indole-3-carboxylic acid | 0.03145 | N-propionylalanine | 0.03875 |
| docosadienoate (22:2n6) | 0.03146 | linoleoyl-docosahexaenoyl-glycerol (18:2/22:6) [2] | 0.03884 |
| nicotinate | 0.0315 | 1-ribosyl-imidazoleacetate | 0.03888 |
| 2-aminoadipate | 0.0316 | hydroquinone sulfate | 0.03898 |
| pantothenate (Vitamin B5) | 0.03163 | oleate/vaccenate (18:1) | 0.03956 |
| lignoceroylcarnitine (C24) | 0.03187 | taurolithocholate 3-sulfate | 0.03987 |
| isoleucylglycine | 0.03194 | sinapate | 0.04025 |
| trimethylamine N-oxide | 0.03203 | 2-linoleoylglycerol (18:2) | 0.04053 |
| N-oleoyl-sphingosine (d18:1/18:1) | 0.03266 | anthranilate | 0.04062 |
| 7-methylguanine | 0.03282 | methylsuccinate | 0.04067 |
| 1-oleoyl-2-linoleoyl-GPC (18:1/18:2) | 0.03288 | N-acetylalanine | 0.04069 |
| margarate (17:0) | 0.03299 | oleoylcholine | 0.04084 |
| coprostanol | 0.033 | N-formylmethionine | 0.04085 |
| valylglutamine | 0.03308 | 1-linoleoyl-GPE (18:2) | 0.04115 |
| 3-hydroxy-3-methylglutarate | 0.03308 | arachidonate (20:4n6) | 0.04123 |
| nicotinamide ribonucleotide (NMN) | 0.0331 | trigonelline (N'-methylnicotinate) | 0.04142 |
| trans-urocanate | 0.03323 | 5,6-dihydrothymine | 0.04158 |
| ribose | 0.03353 | sphingomyelin (d18:1/14:0, d16:1/16:0) | 0.04168 |
| 5-hydroxyindoleacetate | 0.0336 | gamma-glutamylleucine | 0.04176 |
| 2-aminophenol | 0.03386 | choline | 0.0418 |
| phenylalanylalanine | 0.0339 | 2-palmitoleoylglycerol (16:1) | 0.04188 |
| phytosphingosine | 0.03396 | glycerate | 0.04192 |
| 2-hydroxymyristate | 0.03396 | arabonate/xylonate | 0.04218 |
| L-urobilin | 0.03412 | 2-myristoylglycerol (14:0) | 0.04221 |
| isoleucine | 0.03434 | 1-palmitoyl-2-arachidonoyl-GPC (16:0/20:4n6) | 0.0423 |
| pregnenolone sulfate | 0.03447 | formiminoglutamate | 0.0425 |
| LAHSA (18:2/OH-18:0) | 0.03462 | thioproline | 0.04253 |
| 5alpha-pregnan-3beta,20beta-diol monosulfate (2) | 0.03469 | 2-aminophenol sulfate | 0.04291 |
| arachidoylcarnitine (C20) | 0.03469 | 1-stearoyl-GPS (18:0) | 0.04317 |
| phenylalanylglycine | 0.03478 | malonylcarnitine | 0.0433 |
| leucylglycine | 0.03528 | palmitoylcholine | 0.04353 |
| phenethylamine | 0.03532 | 1-methylhistamine | 0.04354 |
| docosapentaenoylcarnitine (C22:5n3) | 0.03536 | palmitoyl dihydrosphingomyelin (d18:0/16:0) | 0.04382 |
| threonylphenylalanine | 0.03546 | fumarate | 0.04395 |
| 2-hydroxynervonate | 0.03574 | felinine | 0.04412 |
| eriodictyol | 0.03592 | 3-(3-hydroxyphenyl)propionate sulfate | 0.04418 |
| undecanedioate (C11-DC) | 0.03593 | 2-oleoylglycerol (18:1) | 0.04438 |
| diaminopimelate | 0.0362 | glucose | 0.04463 |
| histidylalanine | 0.0362 | 3-methylglutaconate | 0.04464 |
| 2-hydroxyarachidate | 0.03659 | glutaminylleucine | 0.04501 |
| leucine | 0.03664 | 1-stearoyl-GPC (18:0) | 0.04506 |
| N-propionylalanine | 0.03668 | carboxyethyl-GABA | 0.04535 |
| deoxycholic acid sulfate | 0.03669 | guanidinoacetate | 0.04567 |
| phosphate | 0.03678 | taurocholenate sulfate | 0.04574 |
| octadecanedioylcarnitine (C18-DC) | 0.03684 | guanine | 0.04585 |
| succinimide | 0.03694 | caprate (10:0) | 0.04607 |
| 3-ureidoisobutyrate | 0.03694 | 1-palmitoyl-2-oleoyl-GPE (16:0/18:1) | 0.04617 |
| D-urobilin | 0.03715 | N-acetylthreonine | 0.04652 |
| I-urobilinogen | 0.03716 | thymine | 0.04656 |
| urate | 0.03717 | N-glycolylneuraminate | 0.04656 |
| 1-palmitoyl-GPI (16:0) | 0.0374 | O-sulfo-L-tyrosine | 0.04663 |
| 7alpha-hydroxycholestenone | 0.03742 | azelate (nonanedioate; C9) | 0.04684 |
| biotin | 0.0375 | 10-nonadecenoate (19:1n9) | 0.04713 |
| indolepropionate | 0.03757 | valylglycine | 0.04741 |
| 3-hydroxysebacate | 0.03777 | nicotinamide ribonucleotide (NMN) | 0.04746 |
| 3b-hydroxy-5-cholenoic acid | 0.03795 | tyrosylglycine | 0.04766 |
| tyrosylglycine | 0.03799 | mevalonate | 0.04778 |
| pregnanolone/allopregnanolone sulfate | 0.03809 | creatine | 0.04782 |
| isovalerylphenylalanine | 0.0381 | 2-palmitoylglycerol (16:0) | 0.04822 |
| 2-hydroxybehenate | 0.03833 | valylglutamine | 0.04832 |
| 1-methylurate | 0.03834 | galacturonate | 0.04838 |
| N-acetylsphingosine | 0.03868 | 5,6-dihydrouridine | 0.04862 |
| linoleoyl ethanolamide | 0.03875 | ribulose/xylulose | 0.04912 |
| phenylalanine | 0.03898 | gamma-tocopherol/beta-tocopherol | 0.04922 |
| 7-hydroxycholesterol (alpha or beta) | 0.03909 | dipicolinate | 0.04923 |
| 3-(3-hydroxyphenyl)propionate | 0.03917 | 1-oleoyl-GPC (18:1) | 0.04923 |
| xylose | 0.03948 | stearoylcholine | 0.04927 |
| valylglycine | 0.0395 | threonylphenylalanine | 0.04932 |
| alpha-CEHC | 0.03987 | oleoyl-linolenoyl-glycerol (18:1/18:3) [2] | 0.04936 |
| o-Tyrosine | 0.03997 | 1-palmitoyl-GPG (16:0) | 0.04946 |
| 1,2-dilinoleoyl-GPE (18:2/18:2) | 0.03998 | ergosterol | 0.04959 |
| N-acetyl-isoputreanine | 0.04008 | linoleoyl ethanolamide | 0.04967 |
| thymine | 0.04024 | histidylalanine | 0.04982 |
| 10-heptadecenoate (17:1n7) | 0.0408 | creatinine | 0.05023 |
| uracil | 0.04087 | diacylglycerol (16:1/18:2 [2], 16:0/18:3 [1]) | 0.05049 |
| 21-hydroxypregnanolone monosulfate (1) | 0.04093 | glycylleucine | 0.0505 |
| carotene diol (3) | 0.04118 | phenylalanylalanine | 0.05097 |
| 3-ketosphinganine | 0.04123 | oleoyl-linoleoyl-glycerol (18:1/18:2) [2] | 0.05097 |
| oleoyl-oleoyl-glycerol (18:1/18:1) [1] | 0.04136 | pyrraline | 0.05105 |
| glycylleucine | 0.04149 | uridine | 0.05131 |
| 3-hydroxybutyrylcarnitine (2) | 0.04172 | palmitoyl-linoleoyl-glycerol (16:0/18:2) [2] | 0.05174 |
| beta-alanine | 0.04173 | ribose | 0.0518 |
| erucoylcarnitine (C22:1) | 0.04174 | N-acetyl-beta-glucosaminylamine | 0.05212 |
| 2-hydroxystearate | 0.04178 | 1-palmitoyl-digalactosylglycerol (16:0) | 0.05223 |
| N-methylalanine | 0.04223 | galactitol (dulcitol) | 0.05228 |
| cis-urocanate | 0.0426 | sphingomyelin (d18:1/17:0, d17:1/18:0, d19:1/16:0) | 0.0523 |
| N-acetyl-beta-glucosaminylamine | 0.04269 | N-linoleoyltaurine | 0.0523 |
| citrulline | 0.04275 | 1,2-dilinolenoyl-galactosylglycerol (18:3/18:3) | 0.05249 |
| 1-methylhistidine | 0.04299 | stearoyl-linoleoyl-glycerol (18:0/18:2) [2] | 0.05253 |
| palmitoleate (16:1n7) | 0.04308 | taurine | 0.05279 |
| 2-hydroxypalmitate | 0.04309 | diacylglycerol (14:0/18:1, 16:0/16:1) [2] | 0.05281 |
| glycerol | 0.04322 | 2'-deoxyuridine | 0.05318 |
| hydroxystearate sulfate | 0.04368 | 1-palmitoyl-2-stearoyl-GPC (16:0/18:0) | 0.05324 |
| asparagine | 0.04374 | 2'-deoxycytidine | 0.05333 |
| 1,2-dilinoleoyl-galactosylglycerol (18:2/18:2) | 0.04385 | linoleoyl-linolenoyl-glycerol (18:2/18:3) [2] | 0.05334 |
| 5alpha-pregnan-3beta,20beta-diol disulfate | 0.04419 | palmitoyl-palmitoyl-glycerol (16:0/16:0) [1] | 0.0534 |
| N-palmitoylserine | 0.04431 | oleoyl-oleoyl-glycerol (18:1/18:1) [2] | 0.05343 |
| pregnen-diol disulfate | 0.0446 | lysylleucine | 0.05347 |
| oleoyl-linoleoyl-glycerol (18:1/18:2) [1] | 0.04473 | linolenoyl-linolenoyl-glycerol (18:3/18:3) [2] | 0.0535 |
| palmitoyl-oleoyl-glycerol (16:0/18:1) [1] | 0.04509 | adipate | 0.05351 |
| palmitoleoyl-linoleoyl-glycerol (16:1/18:2) [1] | 0.04519 | adenosine | 0.05356 |
| diacylglycerol (14:0/18:1, 16:0/16:1) [1] | 0.04536 | ferulate | 0.05357 |
| palmitoyl-palmitoyl-glycerol (16:0/16:0) [1] | 0.04539 | linoleoyl-linoleoyl-glycerol (18:2/18:2) [1] | 0.05367 |
| linoleoyl-linoleoyl-glycerol (18:2/18:2) [1] | 0.04553 | chrysoeriol | 0.0537 |
| stearoyl-linoleoyl-glycerol (18:0/18:2) [2] | 0.04559 | isoleucylglycine | 0.0538 |
| palmitoyl-myristoyl-glycerol (16:0/14:0) [2] | 0.04569 | 2-stearoyl-GPE (18:0) | 0.05393 |
| 1,2-dilinoleoyl-GPC (18:2/18:2) | 0.04572 | 1-linoleoyl-GPC (18:2) | 0.05414 |
| 1-linoleoyl-2-linolenoyl-GPC (18:2/18:3) | 0.04626 | palmitoyl-oleoyl-glycerol (16:0/18:1) [1] | 0.05415 |
| docosapentaenoate (DPA; 22:5n3) | 0.04644 | oleoyl ethanolamide | 0.05425 |
| methylmalonate (MMA) | 0.04644 | secoisolariciresinol | 0.05432 |
| heptadecasphingosine (d17:1) | 0.04651 | palmitoyl-linoleoyl-glycerol (16:0/18:2) [1] | 0.05444 |
| 1-dihomo-linolenylglycerol (20:3) | 0.04666 | palmitoyl-oleoyl-glycerol (16:0/18:1) [2] | 0.05452 |
| palmitoyl-palmitoyl-glycerol (16:0/16:0) [2] | 0.04684 | 1-pentadecanoylglycerol (15:0) | 0.05459 |
| caprate (10:0) | 0.04697 | stearoyl-linolenoyl-glycerol (18:0/18:3) [2] | 0.05464 |
| linoleoyl-arachidonoyl-glycerol (18:2/20:4) [2] | 0.04721 | N-acetylmethionine | 0.05473 |
| 5-dodecenoate (12:1n7) | 0.04721 | cytidine | 0.0551 |
| 5alpha-pregnan-3alpha,20beta-diol disulfate | 0.04727 | oleoyl-linoleoyl-glycerol (18:1/18:2) [1] | 0.05527 |
| retinol (Vitamin A) | 0.04727 | palmitoyl-linolenoyl-glycerol (16:0/18:3) [2] | 0.05535 |
| beta-sitosterol | 0.04754 | sarcosine | 0.05537 |
| hexadecasphingosine (d16:1) | 0.04763 | arabitol/xylitol | 0.05538 |
| 1,2-dilinolenoyl-galactosylglycerol (18:3/18:3) | 0.04765 | succinate | 0.0554 |
| thiamin (Vitamin B1) | 0.04777 | xanthine | 0.05545 |
| glutaminylleucine | 0.04783 | oleoyl-oleoyl-glycerol (18:1/18:1) [1] | 0.05573 |
| oleoyl-oleoyl-glycerol (18:1/18:1) [2] | 0.0479 | linoleoyl-linolenoyl-glycerol (18:2/18:3) [1] | 0.05576 |
| behenoylcarnitine (C22) | 0.04791 | pyridoxine (Vitamin B6) | 0.05576 |
| tetradecanedioate (C14) | 0.04797 | DIMBOA | 0.05578 |
| oleoyl-linoleoyl-glycerol (18:1/18:2) [2] | 0.04836 | sphingomyelin (d18:1/24:1, d18:2/24:0) | 0.05578 |
| ceramide (d18:2/24:1, d18:1/24:2) | 0.04846 | phenylalanylglycine | 0.05582 |
| diacylglycerol (12:0/18:1, 14:0/16:1, 16:0/14:1) [2] | 0.04849 | diacylglycerol (14:0/18:1, 16:0/16:1) [1] | 0.05584 |
| hexadecasphinganine (d16:0) | 0.04865 | diacylglycerol (12:0/18:1, 14:0/16:1, 16:0/14:1) [2] | 0.05585 |
| palmitoyl-linoleoyl-glycerol (16:0/18:2) [1] | 0.04885 | leucylglutamine | 0.0559 |
| oleoyl-arachidonoyl-glycerol (18:1/20:4) [2] | 0.04887 | palmitoleoyl-linoleoyl-glycerol (16:1/18:2) [1] | 0.05606 |
| linoleate (18:2n6) | 0.04897 | N-acetylglucosamine 6-sulfate | 0.05609 |
| arachidoyl ethanolamide (20:0) | 0.04907 | palmitoyl-palmitoyl-glycerol (16:0/16:0) [2] | 0.05614 |
| dihomolinoleate (20:2n6) | 0.04907 | palmitoyl-myristoyl-glycerol (16:0/14:0) [2] | 0.05632 |
| 1-pentadecanoylglycerol (15:0) | 0.04907 | linolenoyl-linolenoyl-glycerol (18:3/18:3) [1] | 0.05671 |
| palmitoyl-oleoyl-glycerol (16:0/18:1) [2] | 0.0492 | propionylglutamine | 0.05695 |
| 3beta-hydroxy-5-cholestenoate | 0.04951 | nicotinate | 0.05713 |
| glycylvaline | 0.0497 | nicotinamide riboside | 0.05749 |
| gamma-tocotrienol | 0.04974 | pyridoxal | 0.05774 |
| alpha-tocotrienol | 0.05009 | nicotinate ribonucleoside | 0.05793 |
| diacylglycerol (14:0/18:1, 16:0/16:1) [2] | 0.05013 | valylleucine | 0.05829 |
| eicosenoylcarnitine (C20:1) | 0.05046 | 1,2-dilinoleoyl-GPE (18:2/18:2) | 0.05839 |
| 15-methylpalmitate (i17:0) | 0.05055 | 1-(1-enyl-oleoyl)-GPE (P-18:1) | 0.0584 |
| linoleoyl-linolenoyl-glycerol (18:2/18:3) [1] | 0.05062 | leucylglycine | 0.05842 |
| gamma-glutamylleucine | 0.05063 | 3-(3-hydroxyphenyl)propionate | 0.05843 |
| secoisolariciresinol | 0.05064 | guanosine | 0.05848 |
| linolenoyl-linolenoyl-glycerol (18:3/18:3) [1] | 0.05104 | erythrose | 0.05872 |
| 1-palmitoylglycerol (16:0) | 0.05121 | palmitoyl sphingomyelin (d18:1/16:0) | 0.05874 |
| tyrosine | 0.05131 | linolenate (18:3n3 or 3n6) | 0.05877 |
| palmitoyl-linolenoyl-glycerol (16:0/18:3) [2] | 0.05134 | inosine | 0.0589 |
| 3beta,7beta-dihydroxy-5-cholestenoate | 0.05163 | 1-methylnicotinamide | 0.05892 |
| chrysoeriol | 0.05173 | 1,2-dilinoleoyl-digalactosylglycerol (18:2/18:2) | 0.05905 |
| diacylglycerol (16:1/18:2 [2], 16:0/18:3 [1]) | 0.05175 | 1-linoleoyl-GPG (18:2) | 0.0591 |
| indolin-2-one | 0.0521 | 1-stearoyl-GPE (18:0) | 0.05913 |
| N-stearoyl-sphingosine (d18:1/18:0) | 0.05237 | 2'-deoxyinosine | 0.05988 |
| 10-hydroxystearate | 0.05253 | 1-oleoyl-GPG (18:1) | 0.06034 |
| stearoyl-linolenoyl-glycerol (18:0/18:3) [2] | 0.05254 | fucose | 0.06058 |
| palmitoyl-linoleoyl-glycerol (16:0/18:2) [2] | 0.05255 | 1,2-dilinoleoyl-GPC (18:2/18:2) | 0.0606 |
| 1-oleoylglycerol (18:1) | 0.0527 | alanylleucine | 0.06069 |
| myristoleate (14:1n5) | 0.05281 | 1-linoleoyl-2-linolenoyl-GPC (18:2/18:3) | 0.06093 |
| oleoyl-linolenoyl-glycerol (18:1/18:3) [2] | 0.0532 | palmitoyl-arachidonoyl-glycerol (16:0/20:4) [2] | 0.06098 |
| N-stearoyl-sphinganine (d18:0/18:0) | 0.05334 | xylose | 0.061 |
| isoursodeoxycholate | 0.05334 | propionylglycine (C3) | 0.06127 |
| alpha-tocopherol | 0.05352 | leucylalanine | 0.06161 |
| cholesterol | 0.05355 | thymidine | 0.06165 |
| linolenoyl-linolenoyl-glycerol (18:3/18:3) [2] | 0.05356 | 1-palmitoyl-2-linoleoyl-digalactosylglycerol (16:0/18:2) | 0.06175 |
| sphingosine | 0.05361 | 1-palmitoleoylglycerol (16:1) | 0.06206 |
| 3beta,7alpha-dihydroxy-5-cholestenoate | 0.0537 | 1-linoleoylglycerol (18:2) | 0.06218 |
| linoleoyl-linolenoyl-glycerol (18:2/18:3) [2] | 0.05391 | kynurenine | 0.06221 |
| eicosanoylsphingosine (d20:1) | 0.05406 | 1-myristoylglycerol (14:0) | 0.06257 |
| octadecenedioate (C18:1-DC) | 0.05412 | hypoxanthine | 0.06259 |
| ximenoyl ethanolamide (26:1) | 0.05413 | 1-stearoyl-GPI (18:0) | 0.06279 |
| 12,13-DiHOME | 0.05423 | nicotinamide | 0.06285 |
| 1-myristoylglycerol (14:0) | 0.05489 | oleoyl-arachidonoyl-glycerol (18:1/20:4) [2] | 0.063 |
| palmitoyl-arachidonoyl-glycerol (16:0/20:4) [2] | 0.05494 | glycerophosphoserine | 0.06311 |
| 9,10-DiHOME | 0.05509 | 1-linolenoylglycerol (18:3) | 0.06311 |
| behenoyl ethanolamide (22:0) | 0.05515 | dihydroferulic acid | 0.0633 |
| ergosterol | 0.05544 | 1-palmitoyl-GPE (16:0) | 0.0635 |
| hexadecenedioate (C16:1-DC) | 0.05547 | 3-hydroxy-3-methylglutarate | 0.06355 |
| nervonoyl ethanolamide (24:1) | 0.05547 | 1-(1-enyl-palmitoyl)-GPE (P-16:0) | 0.06386 |
| 1-linolenoylglycerol (18:3) | 0.05574 | 1-oleoylglycerol (18:1) | 0.06392 |
| 1-palmitoleoylglycerol (16:1) | 0.05593 | N-acetylglucosamine/N-acetylgalactosamine | 0.06395 |
| linoleoyl-linoleoyl-glycerol (18:2/18:2) [2] | 0.05606 | cysteinylglycine | 0.06398 |
| 1-linoleoylglycerol (18:2) | 0.0565 | N-acetylmuramate | 0.0641 |
| N-formylanthranilic acid | 0.05679 | linoleoyl-arachidonoyl-glycerol (18:2/20:4) [2] | 0.06474 |
| lignoceroyl ethanolamide (24:0) | 0.05726 | glycerophosphoinositol | 0.06519 |
| indole | 0.05729 | glycerophosphoglycerol | 0.06544 |
| hexadecadienoate (16:2n6) | 0.05747 | 1-palmitoyl-GPI (16:0) | 0.06571 |
| campesterol | 0.05765 | 1,2-dipalmitoyl-GPC (16:0/16:0) | 0.06577 |
| 6-hydroxynicotinate | 0.05768 | 1,2-dilinoleoyl-galactosylglycerol (18:2/18:2) | 0.06578 |
| serotonin | 0.05813 | 1-stearoyl-GPG (18:0) | 0.06598 |
| ceramide (d18:1/20:0, d16:1/22:0, d20:1/18:0) | 0.05814 | 2'-deoxyguanosine | 0.06602 |
| deoxycholate | 0.05833 | secoisolariciresinol diglucoside | 0.06635 |
| enterolactone | 0.05841 | cysteine | 0.06643 |
| linolenate (18:3n3 or 3n6) | 0.05845 | benzoate | 0.06644 |
| lithocholate | 0.05878 | 1-palmitoylglycerol (16:0) | 0.06695 |
| 13-HODE + 9-HODE | 0.059 | gentisate | 0.06703 |
| sphinganine | 0.05925 | 1-dihomo-linolenylglycerol (20:3) | 0.06724 |
| ceramide (d18:1/14:0, d16:1/16:0) | 0.0593 | stachydrine | 0.06776 |
| carboxyethyl-GABA | 0.05934 | mannose | 0.0704 |
| gamma-tocopherol/beta-tocopherol | 0.05994 | 1-(1-enyl-stearoyl)-GPE (P-18:0) | 0.07115 |
| 2-myristoylglycerol (14:0) | 0.06026 | 1-palmitoyl-2-oleoyl-GPC (16:0/18:1) | 0.07159 |
| pterin | 0.06095 | linoleate (18:2n6) | 0.07164 |
| dihomolinolenate (20:3n3 or 3n6) | 0.06132 | 1-oleoyl-2-linoleoyl-GPC (18:1/18:2) | 0.0719 |
| delta-tocopherol | 0.06168 | 1-palmitoyl-2-linoleoyl-GPE (16:0/18:2) | 0.0719 |
| N-palmitoyl-sphingosine (d18:1/16:0) | 0.06176 | fructose | 0.07334 |
| 2-palmitoleoylglycerol (16:1) | 0.0619 | glycerol | 0.07346 |
| hexadecanedioate (C16) | 0.06246 | vanillate | 0.07472 |
| 2-palmitoylglycerol (16:0) | 0.06257 | 1-oleoyl-GPE (18:1) | 0.07602 |
| N-palmitoyl-sphinganine (d18:0/16:0) | 0.06394 | 1-oleoyl-2-linoleoyl-GPE (18:1/18:2) | 0.07678 |
| 2-oleoylglycerol (18:1) | 0.06424 | arabinose | 0.07723 |
| 2-linoleoylglycerol (18:2) | 0.06426 | 4-hydroxycinnamate | 0.07826 |
| beta-muricholate | 0.06426 | glucuronate | 0.07841 |
| enterodiol | 0.06427 | syringic acid | 0.07887 |
| ceramide (d18:1/17:0, d17:1/18:0) | 0.06446 | 1-palmitoyl-2-palmitoleoyl-GPC (16:0/16:1) | 0.08005 |
| N-(2-hydroxypalmitoyl)-sphingosine (d18:1/16:0(2OH)) | 0.06488 | 1-palmitoyl-2-linoleoyl-GPC (16:0/18:2) | 0.08105 |
| 1-methyl-beta-carboline-3-carboxylic acid | 0.06507 | glycerophosphoethanolamine | 0.08129 |
| glycylisoleucine | 0.06574 | 2-aminophenol | 0.08196 |
| 13-methylmyristate (i15:0) | 0.06583 | 4-guanidinobutanoate | 0.08242 |
| N-palmitoyl-heptadecasphingosine (d17:1/16:0) | 0.06647 | 5-aminovalerate | 0.085 |
